# Dietary supplementation with proanthocyanidins and rutin alleviates the symptoms of type 2 diabetes mice and regulates gut microbiota

**DOI:** 10.3389/fmicb.2024.1513935

**Published:** 2025-01-06

**Authors:** Yue Gao, Binbin Huang, Yunyi Qin, Bing Qiao, Mengfei Ren, Liqing Cao, Yan Zhang, Maozhen Han

**Affiliations:** ^1^School of Life Sciences, Anhui Medical University, Hefei, Anhui, China; ^2^School of Life Sciences, Hefei Normal University, Hefei, Anhui, China; ^3^Microbial Medicinal Resources Development Research Team, Anhui Provincial Institute of Translational Medicine, Hefei, Anhui, China; ^4^School of Public Health, Anhui Medical University, Hefei, Anhui, China

**Keywords:** type 2 diabetes, gut microbiota, rutin, proanthocyanidins, body weight, fasting blood glucose

## Abstract

**Background:**

Obesity and high fasting blood glucose (FBG) resulting from high-fat diets (HFDs) have emerged as significant public health concerns, garnering increasing attention. Recently, gut microbiota has been linked with metabolic diseases such as type 2 diabetes (T2DM), and its mediating role in dietary supplements has been confirmed. Seeking various dietary supplements to lose body weight (BW) and decrease FBG and explaining the underlying mechanism have become the research hotspots in T2DM studies.

**Methods:**

In this study, rutin and proanthocyanidins (PA) were selected as dietary supplements (200 mg/kg × day, oral gavage, 6 weeks) in T2DM mice induced with HFD to assess their efficacy in weight loss, FBG reduction, gut microbiota alterations, and the associated underlying mechanisms.

**Results:**

Our findings indicate that rutin was more effective than PA in relieving inflammation and fat hypertrophy, although both significantly reduced BW and FBG within 2 weeks after the intervention. Analysis of 16S rRNA amplicons revealed substantial alterations in the gut microbial community composition of mice administered with PA and rutin compared to HFD-fed mice. Importantly, several core microbes, particularly a series of probiotics, such as *Akkermansia*, *Lactococcus*, *Odoribacter*, *Faecalibaculum*, and *Roseburia* were identified, which were significantly correlated with the changes in BW and FBG.

**Conclusion:**

Overall, our study highlights that rutin and PA can reduce BW, FBG, and inflammation by modulating the gut microbiota composition, providing novel perspectives for managing and treating weight and FBG concerns in obesity and T2DM patients through dietary supplements in clinical treatment.

## Introduction

Obesity is a chronic metabolic disorder, which significantly elevates the susceptibility to various diseases, including type 2 diabetes (T2DM) (Fryk et al., 2021), fatty liver (Eslam et al., 2020), hypertension (DeMarco et al., 2014; Eslam et al., 2020), myocardial infarction (Yusuf et al., 2005), and cancer (Hopkins et al., 2016). According to the World Health Organization, more than 1 billion people worldwide suffer from obesity and its derived metabolic syndromes including T2DM (World Health Organization., 2021). Consideration of T2DM is a global epidemic characterized by metabolic disorders and harboring many complications caused by genetic background and environmental factors, such as overnutrition (Donath and Shoelson, 2011), and the number of cases could exceed 642 million by 2040 without intervention (Ogurtsova et al., 2017). Hence, our present study focuses on T2DM as a disease model to explore the treatment strategy from the perspectives of microbiome approach and dietary supplementation.

In past decades, previous studies have confirmed that gut microbiota and its metabolites play an important role in the metabolism of the body and are closely associated with metabolic diseases (Fan and Pedersen, 2021). In particular, scientists have proven that the dysbiosis of gut microbiota is an important contributor to the prevalence and development of metabolic diseases such as obesity and T2DM (de Vos et al., 2022; Han et al., 2022; Ley et al., 2006) and proposed several new strategies from the perspectives of dietary, probiotics, and prebiotics for the treatment of metabolic diseases, including T2DM (Asemi et al., 2016; Delzenne et al., 2015; Kim et al., 2018; Yang et al., 2021; Yoo and Kim, 2016). It is noteworthy that various studies have highlighted the influence of diet as a pivotal factor in shaping the taxonomical and functional compositions of gut microbial communities (Sonnenburg and Bäckhed, 2016). Recent findings have underscored the anti-diabetic properties of dietary natural products, which harbor the ability to support the maintenance of gut microbial balance (de Vos et al., 2022). Several dietary natural products, such as 6-shogaol (Jiao et al., 2023), tea polyphenol (Wen et al., 2023), resveratrol (Jeyaraman et al., 2020), and myricetin (Zhao et al., 2022), have demonstrated the ability to modulate gut microbiota, enhance gut integrity, regulate host metabolism, and alleviate obesity and T2DM (Chen et al., 2019; Delzenne et al., 2011). Nowadays, although dietary natural products are promising nutritional interventions with minimal side effects in treating T2DM, exploring more dietary supplements with functional properties and benchmarking the effects of different dietary supplements are urgently needed, and the underlying mechanism should be investigated.

Polyphenols, including tea polyphenols, grape seed proanthocyanidins, and resveratrol, are famous natural dietary compounds that are enriched in plant and human foods. For example, the largest dietary sources of proanthocyanidins (PA) are pulses, grains, nuts, cocoa, tea, wine, and fruits, such as blueberries [(179.8 ± 50.8 ∼331.9 ± 14.0) mg/100 g fresh weight], plums (215.9 ± 50.7 mg/100 g fresh weight), grapes (81.5 ± 15.0 mg/100 g fresh weight), and apples [(47.2 ± 0.6 ∼ 141.0 ± 26.1) mg/100 g fresh weight] (Gu et al., 2004; Salvadó et al., 2015). The function of polyphenols in alleviating the metabolic syndrome associated with obesity has been confirmed (Tomás-Barberán et al., 2016). PA has been suggested to account for a significant portion of polyphenols consumed in the Western diet because of its ubiquity (Prior and Gu, 2005). Moreover, research studies have revealed that approximately 11% of PA can be identified in the feces after consuming PA daily for 10 days. This finding suggests the gastrointestinal health benefits of the stable presence of ingested PA in the gut (Choy et al., 2013). Several studies have demonstrated the function of PA supplementation and explored the mechanism relying on gut microbiota in-depth (Redondo-Castillejo et al., 2023), providing potential for the prevention and treatment of gut microbiota disorders (Campos et al., 2021; Gonzalez-Abuin et al., 2015; Hameed et al., 2020; Liu W. et al., 2017). More importantly, current toxicological studies showed that PA have no observable toxicity to humans when used appropriately (Zeng et al., 2020). In contrast, rutin is famous as one of the natural polyphenol flavonoids that can be obtained from diverse plants, such as buckwheat (up to 396 mg/100 g), amaranth leaves [up to 24.5 g/kg (dry weight)], elderflower tea (around 10.9 g/kg of dry flowers’ weight), unfermented rooibos tea (about 1.69 mg/g), and acacias (Gullón et al., 2017). It has been reported to have a wide range of biological activities and pharmacological effects, including reducing fasting blood glucose (FBG) and body weight (BW), anti-inflammatory and antioxidant properties, and neuroprotection (Ghorbani, 2017; Kamalakkannan and Prince, 2006; Ola et al., 2015; Tung et al., 2021; Yeh et al., 2014; Yuan et al., 2017). Increasing evidence confirms that rutin is a type of naturally occurring flavonoid, which is widely found in vegetables and fruits; it has beneficial effects on diabetes as a dietary supplement to improve glycemic control, lipid profile, and antioxidant status with no apparent toxicity and poor bioavailability in rodents (Bazyar et al., 2023; Ghorbani, 2017; Liu Q. et al., 2017; Valentová et al., 2014), suggesting that rutin is a potential substrate or dietary supplement in clinical trial. Notably, the pharmacological properties of rutin are gradually explored, such as substantial anti-inflammatory and antioxidant effects. However, no evidence could confirm that rutin can modulate the gut microbiota in high-fat diet (HFD)-induced T2DM mice. Besides, few studies focused on the comparison of the effects of PA and rutin (both plant polyphenols and available dietary supplements) on weight loss and reduction in FBG levels and their comparative abilities to repair the gut microbiota.

Therefore, to decipher and compare the possible mechanisms by which dietary supplementation with PA or rutin reduces BW and FBG levels in HFD-induced T2DM mice by modulating the gut microbiota, we established a T2DM mouse model derived from obesity induced by HFD and subsequent oral administration of PA or rutin (both 200 mg/kg × day), comparing them with normal and positive control groups. During the intervention process, the BW and FBG levels of mice were measured with a high-frequency strategy to directly revel the effects of rutin and PA. Whereas the feces of mice were collected at two time points, namely, the time of successful modeling and 6 weeks after the intervention (week 10). The V3-V4 region of the 16S rRNA was sequenced to investigate the alterations of gut microbiota. Besides, the oral glucose tolerance tests (OGTT) and intraperitoneal glucose tolerance tests (IPITT) were conducted at the end of the intervention and the pancreatic (PAN), liver, and adipose tissues of mice were collected for weighing and hematoxylin and eosin (H&E) staining to compare the effects of rutin and PA. Our results showed that rutin and PA harbor the ability to significantly lose BW and decrease FBG levels in T2DM mice. The results of 16S rRNA amplicon analysis revealed that the gut microbiota underwent dynamic changes in the groups administered orally with PA and rutin. Particularly, our results identified several probiotics, such as *Akkermansia*, *Lactococcus*, *Odoribacter*, *Faecalibaculum*, and *Roseburia*, which were significantly associated with the changes in BW and FBG. Moreover, the results of H&E staining suggested that rutin is more effective than PA in relieving inflammation and fat hypertrophy.

In conclusion, our study demonstrated that the function and mechanism of rutin and PA in anti-inflammatory, BW loss, and FBG level decrease rely on the modulation of the gut microbiota. Furthermore, the therapeutic effects of rutin are better than those of PA. Finally, the application scenarios in which they maximize their function may differ. Overall, our results provide new insights from the perspectives of the gut microbiome and dietary supplements into the clinical management and treatment of BW and FBG in patients with obesity and T2DM.

## Materials and methods

### Experimental animals and treatments

**FIGURE 1:**
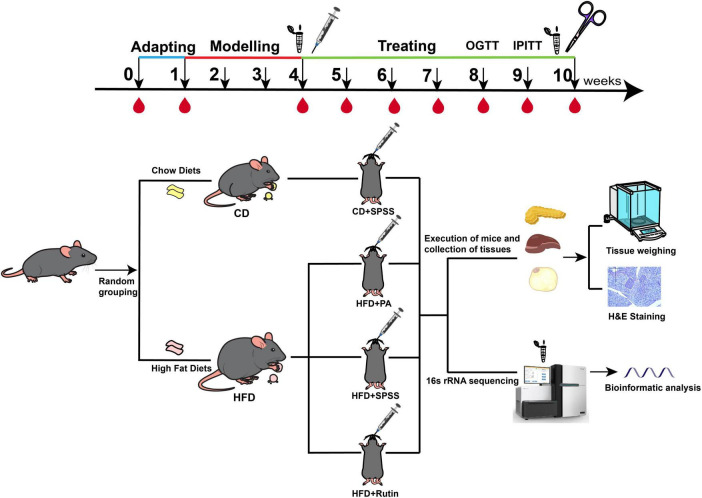
Experimental design and procedure for comparing the effects and underlying mechanisms of PA and rutin in reducing BW and decreasing FBG levels. C57BL/6 mice were acclimated for 1 week, designated as week 0. The T2DM model was induced by continuous HFD feeding over 4 weeks. Subsequently, the T2DM mice were divided into three groups: HFD+SPSS, HFD+PA, and HFD+Rutin groups. The intervention experiment lasted 6 weeks. BW and FBG were measured weekly across the entire period of the experiment. During the period of the model construction model and intervention experiment, fecal samples were collected from each mouse at the time of successful model construction (week 4) and the end of intervention (week 10) and stored at −80°C. The PAN, liver, and adipose tissues were collected and soaked in 4% paraformaldehyde for downstream analysis, including H&E staining and weighing. In addition, OGTT was performed at week 8 and IPITT at week 9.

A total of 20 4-week-old male specific-pathogen-free (SPF) C57BL/6 mice were purchased from the Experimental Animal Center of Anhui Medical University (Hefei, China). Five mice were housed per cage with free access to food and sterile drinking water in a temperature-controlled room (21°C ± 2°C) under a 12-h light-dark cycle. Initially, the mice were acclimated to the new environment by being fed a standard chow diet (CD) for 1 week (defined as week 0). Following the adaptation period, the mice were randomly divided into two groups based on CD (13.5% of energy from fat; LabDiet 5001; LabDiet, USA) and HFD (45% of energy from fat; D12451; Research DIETS, USA): CD (*N* = 5) and HFD (*N* = 15) groups. Subsequently, by feeding the mice HFD for 4 weeks and measuring FBG after giving the mice overnight fasting for 12 h, with FBG > 9.5 mmol/L, the T2DM mouse model was successfully constructed (Cheng et al., 2019; Pang et al., 2016; Yu et al., 2019), the HFD group was further subdivided into three groups and there was no significant difference in BW and FBG among these three groups (*p* > 0.05, *t*-test). Prior research investigating the efficacy of PA in treating obesity involved a 7-week administration of a 300 mg/kg dosage (Liu W. et al., 2017). In contrast, other studies employed varying dosages (75, 150, and 300 mg/kg) over a 4-week period (Liu et al., 2022) and intervened with rutin (200 mg/kg) in HFD-fed mice for 4 weeks was found to be effective in combating obesity and improving lipid metabolism (Liu Q. et al., 2017). Considering these variables, we administered a daily oral dose of 200 mg/kg/day of PA (>95% purity; Aladdin; HFD+PA group), another received 200 mg/kg/day of rutin (>95% purity; Aladdin; HFD+Rutin group), while the third group was given stroke-physiological saline solution (SPSS) as the negative control (HFD+SPSS group). Meanwhile, the CD mice were given an equal volume of SPSS as the normal control (CD+SPSS group). At week 10, the mice were euthanized through cervical dislocation under isoflurane inhalation anesthesia. The PAN, liver tissue, and adipose tissue were collected and soaked in 4% paraformaldehyde. Fecal samples were collected at the time of successful construction of the T2DM mouse model at week 4 of the experiment and at the time of successful treatment at week 10 of the experiment, and the fecal samples were stored at −80°C (Figure 1). Ethical approval for the animal experiments was obtained from the Experimental Animal Ethics Committee of Anhui Medical University (approval no: LLSC20210503).

### Measurement of BW, FBG, OGTT, and IPITT

The BW and FBG levels of each mouse were measured weekly in accordance with the description of Hosomi et al. (2022) to estimate the therapeutic effects of PA and rutin in reducing BW and decreasing FBG levels. The FBG level was measured by sampling blood from the tail tip with a Roche Accu-Chek Performa (Roche Diagnostics, Mannheim, Germany) (Dhatt et al., 2011). Furthermore, the changes in BW and FBG during the intervention process were calculated using the following formulas:


(1) ΔBW=B⁢W¯-10⁢wB⁢W¯4⁢w

(2) ΔFBG=F⁢B⁢G¯-10⁢wF⁢B⁢G¯4⁢w

where $\bar{B\,W}$_10w_ and $\bar{B\,W}$_4w_ represent the average BW of five mice at weeks 10 and 4, respectively; $\bar{F\,B\,G}$_10w_ and $\bar{F\,B\,G}$_4w_ represent the average FBG of five mice at weeks 10 and 4, respectively; and ΔBW and ΔFBG represent the value of weight loss and blood glucose decrease between weeks 10 and 4, respectively.

Additionally, to evaluate the metabolic status of mice and determine the therapeutic effects of rutin and PA, OGTT and IPITT experiments were performed at weeks 8 and 9, respectively. For the OGTT at week 8, mice underwent a 16-h overnight fast, while for the IPITT at week 9, a 6-h fast was employed. The end time of the measurement of FBG for each mouse was defined as its start time (0 min). Then, the FBG levels in the OGTT and IPITT experiments were measured at 15, 30, 60, and 120 min after oral administration of D-(+)-glucose (1.5 g/kg-BW) and intraperitoneal injection of insulin (0.5 U/kg-BW), respectively. The results of OGTT and IPITT were presented by the area under the curve (AUC) (Yao et al., 2021).

### Weighing and H&E staining of tissues

At the end of the intervention experiment, PAN, liver, and adipose tissues including inguinal adipose tissue (IAT) and gonadal adipose tissue (GAT), of mice were collected and kept for weighing and H&E staining. These tissues were first washed with phosphate-buffered saline (PBS), and then their weight was measured and recorded. Next, H&E staining was conducted by the procedure of Zhang et al. (2020). Eventually, the cell nucleus of these tissues was stained with hematoxylin, and the cytoplasm was stained with eosin. The results of H&E staining were observed and visualized with a microscope (Leica DM2500).

### Collection of fecal samples and 16S rRNA amplicon sequencing

During the entire experiment, the fecal samples were collected at two time points: the time of successful model construction (week 4) and the end of intervention (week 10). Thus, a total of 30 fecal samples were collected. The total DNA was extracted according to the instruction of the PowerSoil DNA Isolation Kit (MoBio, USA) and then all extracted DNA was dissolved in TE buffer and stored at −20°C. Next, the eligible DNA of samples was used as a PCR template for obtaining the V3-V4 hypervariable region of the 16S rRNA gene of microbes. Specifically, 5–50 ng DNA was used as a template for amplifying the V3-V4 amplicon by using the forward (5′-CCTACGGRRBGCASCAGKVRVGAAT-3′) and reverse primers (5′-GGACTACNVGGGTWTCTAATCC-3′). Indexed adapters were added to the ends of 16S rDNA amplicons via limited cycle PCR and the sequencing library was constructed using the MetaVxTM Library Preparation kit. DNA libraries were verified and quantified by an Agilent 2100 Bioanalyzer (Agilent Technologies, Palo Alto, CA, USA) and Qubit^®^ 2.0 (Applied Biosystems, Carlsbad, CA, USA). All sequencing reactions were performed on the Illumina MiSeq platform with a paired-end sequencing strategy by GENEWIZ (Inc., South Plainfield, and NJ, USA).

### Bioinformatic analysis

In this study, 16S rRNA amplicon analysis was conducted in QIIME2 (version: 2021.11) platform (Bokulich et al., 2018; Hall and Beiko, 2018). First, quality control, denoise, chimera removal, and splicing of raw paired-end sequencing reads were performed with the DADA2 module of QIIME2. Second, the representative sequences of microbe features were selected against the trained classifier silva-138-99-nb-classifier.qza by using the command ‘rep-seqs.qza’, and then the taxonomical annotations of these representative sequences were annotated with the command ‘taxonomy. qza’. The relative abundance of each taxa was calculated and summarized in a table and then the taxonomical compositions of the gut microbiota at phylum, class, order, family, genus, and species levels were summarized on R (Prevel et al., 2022).

Furthermore, based on the high quality of 16S rRNA genes and their abundances, PICRUSt2 (version: 2.5.0) was applied to investigate the dynamic changes of functional composition of gut microbial communities during the intervention experiment (Douglas et al., 2020). Two output documents, namely KO_predicted.tsv and pathway_predicted.tsv, were used to explore the alterations of functional traits of gut microbial communities. Besides, to obtain an in-depth understanding of the alterations of gut microbiota, Linear discriminate analysis effect size (LEfSe) (Segata et al., 2011) was applied to identify biomarkers among the CD+SPSS, HFD+SPSS, HFD+PA, and HFD+Rutin groups based on the composition of gut microbiota at week 10. The threshold for the logarithmic LDA score for discriminative features was set to 3.

Additionally, the genera with significant differences (*p* < 0.05, *t*-test) were identified based on the taxonomical compositions of gut microbial communities at weeks 4 (mPA) and 10 (cPA) with the treatment of PA, and those between weeks 4 (mRutin) and 10 (cRutin) with the treatment of rutin. The genera with significant differences presented in the intervention process of PA and rutin were defined as key microbes. Based on the compositions of gut microbial communities of CD+SPSS, HFD+SPSS, HFD+PA, and HFD+Rutin groups, the correlations among genera were calculated using the “corr.test” function of the “psych” package of R, and the corresponding co-occurrence network was visualized in Cytoscape (version: 3.6.1).

In particular, by the calculation formula of Euclidean distance between two points, a novel strategy was proposed to quantitatively illustrate the shift of the gut microbiota between two different groups in this study. Specifically, based on the composition of gut microbiota, principal coordinate analysis (PCoA) was first conducted with the “dudi.pca” function of the “vegan” package, and the values of PCo1 and PCo2 of each sample were obtained. Next, the average values of PCo1 and PCo2 in each group were calculated and defined as the coordinates of the center point of each group. Afterward, the distance of two center points was calculated as described using Formula 3, and it represents the offset of the gut microbial communities between two groups (GM_*offset*_).


(3) GM=o⁢f⁢f⁢s⁢e⁢t⁢(g⁢1-g⁢2)(P⁢C⁢o⁢1g⁢1¯-P⁢C⁢o⁢1g⁢2¯)2+(P⁢C⁢o⁢2g⁢1¯-P⁢C⁢o⁢2g⁢2¯)2

where $\bar{P\,C\,o\,1}$_*g*_*_*n*_* and $\bar{P\,C\,o\,2}$_*g*_*_*n*_* represent the average values of PCo1 and PCo2 in the gut microbiota of group *n*, respectively, and GM_*offset(g1–g2)*_ represents the offset of gut microbial communities between group 1 and group 2.

### Statistical analysis

Statistical analysis was performed mainly on the R platform (version: 4.2.2) and GraphPad Prism software (version: 8.0.2, USA). Shannon and Simpson indices were calculated using the “estimate_richness” function of “phyloseq” package and selected to estimate the changes of α-diversity of gut microbial communities with the Kruskal-Wallis test. Based on the taxonomical composition of microbial communities at the genus level, the Bray–Curtis dissimilarities were calculated and compared with the “anosim” function of the “vegan” package. PCoA was performed and visualized with the “dudi.pca” function of “ade4” and “ggplot2” packages, respectively. The heatmaps presented in this study were visualized with the “pheatmap” package.

## Results

### Verified function of PA and rutin in reducing BW and decreasing FBG in T2DM mice

**FIGURE 2:**
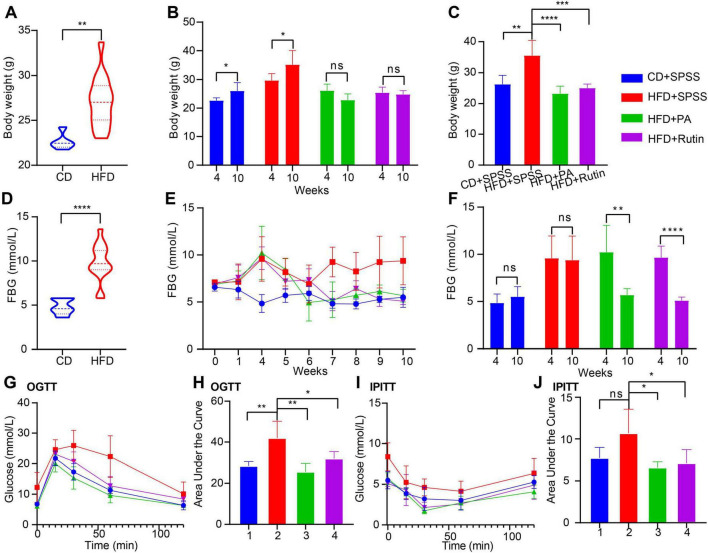
Prompt weight loss and decrease blood glucose in T2DM mice by oral gavage of PA or rutin. **(A)** The BW of CD and HFD groups has a significant difference before the oral administration of PA and rutin (week 4). **(B)** The intervention experiment with oral gavage of PA or rutin to T2DM mice showed that the BW of HFD+PA and HFD+Rutin groups had no significant differences between weeks 10 and 4, respectively, whereas that of CD+SPSS and HFD+SPSS groups significantly increased. **(C)** After oral administration of PA or rutin, the BW of HFD+PA and HFD+Rutin groups were both significantly lower than that of CD+SPSS and HFD+SPSS groups at week 10. **(D)** The FBG levels of CD and HFD groups had a significant difference at week 4. **(E)** Dynamic changes in the FBG of CD+SPSS, HFD+SPSS, HFD+PA, and HFD+Rutin groups across the entire experiment were shown. **(F)** After oral administration of PA or rutin, the FBG levels of HFD+PA and HFD+Rutin groups were significantly lower than those of CD+SPSS and HFD+SPSS groups at week 10. **(G)** OGTT showed the dynamic changes in FBG of CD+SPSS, HFD+SPSS, HFD+PA, and HFD+Rutin groups. **(H)** The AUC values of CD+SPSS and HFD+SPSS groups were significantly higher than those of HFD+PA and HFD+Rutin groups according to OGTT. **(I)** IPITT showed the dynamic changes in the FBG of CD+SPSS, HFD+SPSS, HFD+PA, and HFD+Rutin groups. **(J)** The AUC values of CD+SPSS and HFD+SPSS groups were significantly higher than those of HFD+PA and HFD+Rutin groups according to IPITT.

Several previous studies have demonstrated that PA has the potential to lose BW and decrease FBG (Bertoia et al., 2016), possibly through modulating gut microbiota. However, the function and underlying mechanism of rutin, a plant polyphenol, in obesity and T2DM remains elusive and needs further verification. Hence, a T2DM model of C57BL/6 mice with HFD for 4 weeks was constructed, and the intervention experiment was performed for 6 weeks to clarify whether rutin can alter BW and FBG in patients with obesity and T2DM. As shown in Figure 2A, the BW of the HFD group was significantly higher than that of the CD group at the end of the model construction (all *p* < 0.01, *t*-test). Subsequently, the T2DM mice were orally administrated with PA or rutin for 6 weeks, and the results showed that the BW of HFD+Rutin and HFD+PA groups underwent significant differences across the entire process (Figure 2B and Supplementary Table 1).

On the one hand, the BW of the CD+SPSS and HFD+SPSS groups at week 10 (CD+SPSS: 26.00 ± 2.92 g, HFD+SPSS: 35.20 ± 4.87 g) were significantly higher than that at week 4 (CD+SPSS: 22.65 ± 0.95 g, HFD+SPSS: 29.68 ± 2.40 g, all *p* < 0.05, *t*-test, Figure 2B). However, the BW of HFD+PA and HFD+Rutin groups at week 10 (HFD+PA: 23.00 ± 2.45 g, HFD+Rutin: 24.80 ± 1.30 g) were not significantly higher than that at week 4 (HFD+PA: 26.12 ± 2.26 g, HFD+Rutin: 25.39 ± 1.98 g, all *p* > 0.05, *t*-test, Figure 2B). On the other hand, the BW of the mice fed with HFD was significantly higher than that of the mice fed with CD during the intervention process, particularly at week 10 (*p* < 0.01, ordinary one-way ANOVA, Figure 2C). The BW of the HFD+PA (23.00 ± 2.45 g) and HFD+Rutin groups (24.80 ± 1.30 g) was significantly lower than that of the HFD+SPSS (35.20 ± 4.87 g) group (all *p* < 0.001, ordinary one-way ANOVA, Figure 2C). On the contrary, no significant differences were found among the CD+SPSS (26.00 ± 2.92 g), HFD+PA, and HFD+Rutin groups at week 10 (all *p* > 0.05, ordinary one-way ANOVA, Figure 2C).

Besides, we calculated ΔBW and ΔFBG values to estimate the dynamic changes in BW and FBG for CD+SPSS, HFD+SPSS, HFD+PA, and HFD+Rutin groups and highlight the effects of rutin and PA. The results showed that the BW of the HFD+SPSS group (ΔBW_(*HFD*+*SPSS*)_ = 5.52 g) increased by 2.17 g (39.3%) compared with that of the CD+SPSS group (ΔBW_(*CD*+*SPSS*)_ = 3.35 g). Importantly, we observed that the BW of HFD+PA and HFD+Rutin groups both decreased (ΔBW_(*HFD*+*Rutin*)_ = −0.594 g and ΔBW_(*HFD*+*PA*)_ = −3.116 g) at the end of the intervention experiment. These results suggested that PA and rutin can effectively decrease the BW of T2DM mice, and PA’s effect on reducing weight is better than that of rutin.

Similarly, the FBG levels of the CD+SPSS, HFD+SPSS, HFD+PA, and HFD+Rutin groups were monitored during the entire experiment, and the results showed that the FBG levels of HFD+PA and HFD+Rutin groups significant differed (Supplementary Table 2). Specifically, the FBG levels of the HFD group were significantly higher than that of the CD group at the pre-intervention stage (week 4, *p* < 0.001, *t*-test, Figure 2D). After oral gavage of PA and rutin was administered, the FBG levels of the HFD+PA and HFD+Rutin groups both subsequently decreased, and they can return to a similar level as those in the CD+SPSS group, compared with those in the HFD+SPSS group (Figure 2E). Furthermore, the statistical results of FBG levels among these four groups between weeks 4 and 10 proved that PA and rutin can decrease the FBG levels of T2DM mice (Figure 2F). To estimate the effects of PA and rutin in decreasing FBG levels, we quantified the changes in FBG levels between weeks 10 and 4 for these four groups (ΔFBG_(*HFD*+*SPSS*)_ = −0.2, ΔFBG_(*CD*+*SPSS*)_ = 0.66, ΔFBG_(*HFD*+*Rutin*)_ = −4.56, ΔFBG_(*HFD*+*PA*)_ = −4.54), respectively. The results suggested that the ability of PA and rutin to decrease FBG levels did not show significant differences.

Additionally, to explore the effects of PA and rutin on glucose metabolism, OGTT, and IPITT were conducted at weeks 8 and 9, respectively. The OGTT results revealed that the FBG levels of the HFD+SPSS group at 0, 15, 30, 60, and 120 min were significantly higher than those of the HFD+PA, HFD+Rutin, and CD+SPSS groups (all *p* < 0.05, *t*-test, Figure 2G) at the same time points during the test process. Meanwhile, the AUC of the HFD+SPSS group was significantly higher than that of the HFD+PA, HFD+Rutin, and CD+SPSS groups (all *p* < 0.05, *t*-test, Figure 2H). Besides, we found that the AUC of the HFD+Rutin group was higher than that of the HFD+PA group (Figure 2H). The results of IPITT showed a similar pattern and trend to those of OGTT (Figures 2I, J). Overall, these results demonstrated that administering oral gavage of dietary supplements, namely, PA and rutin, to T2DM mice can restore the ability to modulate blood glucose levels and thereby lose BW and decrease FBG levels.

### Recovery of T2DM mice tissues after oral administration of PA or rutin from a histopathological perspective

**FIGURE 3:**
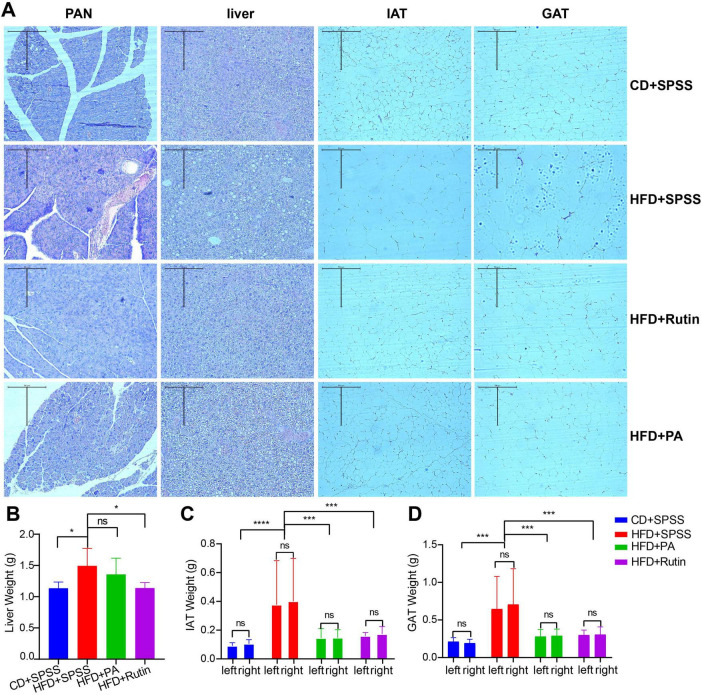
Estimation of histopathological effects of PA and rutin in reducing weight and decreasing blood glucose levels. **(A)** Four kinds of tissues, namely, PAN, liver tissue, IAT, and GAT, were collected and used for H&E staining (10 × 10) for CD+SPSS, HFD+SPSS, HFD+PA, and HFD+Rutin groups. The weight of panel **(B)** liver tissue, **(C)** IAT, and **(D)** GAT among CD+SPSS, HFD+SPSS, HFD+PA, and HFD+Rutin groups were measured and compared to explore the effects of PA and rutin on T2DM mice.

To explore the effects and underlying mechanisms of PA and rutin in reducing BW and decreasing FBG, four kinds of tissues were collected, including PAN, liver tissue, IAT, and GAT. Histopathological examination using H&E staining was conducted, and the weight of these tissues was measured for the CD+SPSS, HFD+SPSS, HFD+PA, and HFD+Rutin (Figure 3). The results showed that the tissue histopathology of different groups changed dramatically, revealing the recovery of function in the tissues after the treatment of PA and rutin. The H&E staining results showed that the cell nuclei deviated, more islet cells degenerated, the arrangement of islet cells was disordered, and intracytoplasmic vesicular structures increased in the physiological tissues of the HFD+SPSS group in comparison with that of the CD+SPSS group (Figure 3A). The liver tissues of the HFD+SPSS group showed obvious fat infiltration and contained many fat vacuoles in the visual field. In particular, fatty degeneration was found in the hepatocytes. By contrast, the hepatocytes of the CD+SPSS group were neatly arranged and structurally intact with clear cell borders and the cell nuclei were in the center of the cells (Figure 3A). The tissue histopathological changes revealed the damage to the physiological function of T2DM mice.

After oral gavage of PA and rutin was administered to T2DM mice for 6 weeks, we were surprised to find that histopathology transformation occurred in the HFD+PA and HFD+Rutin groups. For example, the number of focal vacuolar lesions of islet cells and degenerated islet cells in the HFD+PA group decreased in comparison with that in the HFD+SPSS group (Figure 3A). Furthermore, the islet cells in the HFD+Rutin group showed a neat arrangement, abundant and uniform cell cytoplasm, the disappearance of vacuolar degeneration in the islet cells, and significant improvement in the degree of lesions (Figure 3A). Besides, although the hepatocytes were more structurally intact, the degree of fatty degeneration greatly reduced, and the number of fat vacuoles in the liver of the HFD+PA and HFD+Rutin groups significantly decreased compared with that in the HFD+SPSS group. Fat vacuoles were still present in the liver of these two groups and the proportions in the HFD+PA group were higher than in the HFD+Rutin group. Moreover, the tissue histopathological structures of the HFD+Rutin group were more similar to those of the CD+SPSS group (Figure 3A). These results showed that the oral gavage of PA and rutin in T2DM mice can recover the function of tissues. However, the recovery effectiveness of PA and rutin differ.

Additionally, the results of weight measurement of the liver tissue, IAT, and GAT among the CD+SPSS, HFD+SPSS, HFD+PA, and HFD+Rutin groups showed that the effects on the weight loss of tissues with the treatment of PA and rutin remarkably differed (Supplementary Table 3). Although the liver weight of the HFD+PA (1.36 ± 0.26 g) and HFD+Rutin (1.14 ± 0.09 g) groups decreased compared with that of the HFD+SPSS group (1.49 ± 0.28 g), a significant difference was observed between these two groups (*p* < 0.05, *t*-test), compared with the HFD+PA and HFD+SPSS groups (Figure 3B). In terms of IAT and GAT, the weight of their left and right parts was measured and compared among CD+SPSS, HFD+SPSS, HFD+PA, and HFD+Rutin groups. The results showed that the IAT and GAT weight of the HFD+PA (IAT: 0.146 ± 0.057 g; GAT: 0.284 ± 0.088 g) and HFD+Rutin (IAT: 0.166 ± 0.039 g; GAT: 0.302 ± 0.081 g) groups significantly decreased (IAT: 0.389 ± 0.285 g; GAT: 0.676 ± 0.431 g, all *p* < 0.0001, ordinary one-way ANOVA) in comparison with that of the HFD+SPSS group; no remarkable differences were found in these two groups and with the CD+SPSS group (Figures 3C, D). These results suggested that PA and rutin can decrease the weight of the liver tissue, IAT, and GAT, and the effects of rutin are better than those of PA.

Together, the comparison of histopathological changes and tissue measurements showed that PA and rutin by oral gavage in T2DM mice significantly altered the conformation of the tissue structure and transitioned it from a pathological to a normal state. Moreover, the effects of rutin on fatty metabolism and the weight and morphological structures of the liver were more remarkable than those of PA. Hence, the therapeutic effects of rutin were speculated to be better than those of PA from the perspective of histopathology.

### The significant shift in taxonomical structure of gut microbial communities of T2DM mice after oral gavage of PA and rutin

**FIGURE 4:**
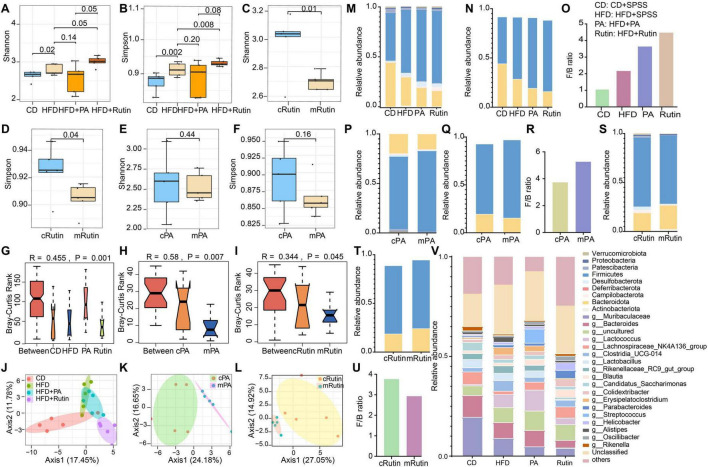
Significant alteration in the taxonomical compositions of gut microbial communities of T2DM mice after oral gavage of PA and rutin. The α diversities, including **(A)** Shannon and **(B)** Simpson indices, of gut microbial communities between the CD+SPSS, HFD+SPSS, HFD+PA, and HFD+Rutin groups were compared. The α diversities, including **(C)** Shannon and **(D)** Simpson indices, of gut microbial communities between mRutin and cRutin groups were compared. The α diversities, including **(E)** Shannon and **(F)** Simpson indices, of gut microbial communities between mPA and cPA groups were compared. The significant differences in the composition of gut microbial communities **(G)** between CD+SPSS, HFD+SPSS, HFD+PA, and HFD+Rutin groups at week 10; **(H)** between pre-intervention (mPA, week 4) and post-intervention (cPA, week 10) groups intervened with PA; and **(I)** between pre-intervention (mRutin, week 4) and post-intervention (cRutin, week 10) groups intervened with rutin were compared by their Bray–Curtis dissimilarity. Principal coordinate analyses **(J)** among CD+SPSS, HFD+SPSS, HFD+PA, and HFD+Rutin groups; **(K)** between cPA and mPA groups; and **(L)** between cRutin and mRutin groups were conducted. **(M)** The taxonomical structure of gut microbial communities for CD+SPSS, HFD+SPSS, HFD+PA, and HFD+Rutin groups was profiled at the phylum level. **(N)** The relative abundances of *Firmicutes* and *Bacteroidota* in CD+SPSS, HFD+SPSS, HFD+PA, and HFD+Rutin groups were visualized. **(O)** The ratios of *Firmicutes* and *Bacteroidota* of CD+SPSS, HFD+SPSS, HFD+PA, and HFD+Rutin groups were calculated and visualized. **(P)** The taxonomical structure of gut microbial communities for cPA and mPA groups was profiled at the phylum level. **(Q)** The relative abundances of *Firmicutes* and *Bacteroidota* in cPA and mPA groups were visualized. **(R)** The ratios of *Firmicutes* and *Bacteroidota* in cPA and mPA groups were visualized. **(S)** The taxonomical compositions of gut microbial communities in cRutin and mRutin groups were profiled at the phylum level. The relative abundances of *Firmicutes* and *Bacteroidota* in panel **(T)** cRutin and mRutin groups were visualized. **(U)** The ratios of *Firmicutes* and *Bacteroidota* in cRutin and mRutin groups were calculated and displayed. **(V)** The top 20 genera were selected and visualized to display the alterations in the taxonomical compositions of gut microbial communities at the genus level.

To investigate the alterations of gut microbiota of T2DM mice caused by PA and rutin and explore the in-depth mechanism, the fecal samples of T2DM mice were collected, the meta-DNA of these samples was extracted, and the V3-V4 region of 16S rRNA was sequenced. Thus, a total of 1,658,576 high-quality sequences were obtained from 30 samples, and the 16S rRNA amplicon dataset was analyzed with the DADA2 tool within QIIME2. The result of the sparsity curve analysis suggested that the sequencing depth was sufficient, and most microbes in each sample were captured (Supplementary Figure 1). Next, the results of α diversity analysis showed that the Shannon and Simpson indices in the gut microbiota of the HFD+Rutin group significantly increased in comparison with those of the HFD+SPSS group (*p* < 0.05, *t*-test, Figures 4A, B). Meanwhile, the Shannon and Simpson indices in the gut microbiota of the HFD+PA group did not significantly differ from that of the HFD+SPSS group (*p* > 0.05, *t*-test, Figures 4A, B). To further illustrate the dynamic changes of α diversities, the α diversities of gut microbial communities between the pre-intervention (mRutin, week 4) and post-intervention (cRutin, week 10) were compared. Similarly, the α diversities in the gut microbial communities of T2DM mice significantly increased with the oral gavage of rutin within 6 weeks (*p* < 0.05, *t*-test, Figures 4C, D), whereas no significant difference was found after the oral administration of PA (mPA and cPA groups, Figures 4E, F). These results suggested that the oral gavage of rutin (200 mg/kg × day) to T2DM mice can significantly alter the α diversity of gut microbial communities, but not PA.

Subsequently, the differences in gut microbial communities among the CD+SPSS, HFD+SPSS, HFD+PA, and HFD+Rutin groups were verified. The results showed that the taxonomical compositions of gut microbial communities at the genus level in the HFD+PA and HFD+Rutin groups significantly differed in comparison with those in the HFD+SPSS group (ANOVA, *p* < 0.05, Figure 4G) at the end of the intervention. Importantly, significant differences were presented in the gut microbial communities of T2DM mice during the process of intervention with PA or rutin (ANOVA, all *p* < 0.05, Figures 4H, I). Moreover, PCoA showed obvious separations in the gut microbial communities of the CD+SPSS, HFD+SPSS, HFD+PA, and HFD+Rutin groups (Figure 4J) and pre-intervention and post-intervention of PA (Figure 4K) and rutin (Figure 4L). These results suggested that oral gavage of rutin (200 mg/kg × day) and PA (200 mg/kg × day) to T2DM mice can significantly change the taxonomical compositions of gut microbial communities in T2DM mice.

Particularly, to further estimate the abilities of PA and rutin to modulate the taxonomical composition of gut microbial communities, the offset value between the two groups was calculated using Formula 3. The results demonstrated that the offset of gut microbial communities between the CD+SPSS and HFD+SPSS groups was 6.68 [GM_*offset*(*CD–HFD*)_ = 6.68], which suggested that the taxonomical compositions of gut microbial communities of the HFD+SPSS group were changed compared to those of the CD+SPSS group. Similarly, the offset values of gut microbial communities among different groups were calculated, and the following values were obtained: GM_*offset*(*HFD*+*PA–HFD*+*SPSS*)_ = 1.79, GM_*offset*(*HFD*+*Rutin–HFD*+*SPSS*)_ = 7.39, GM_*offset*(*cPA*–*mPA*)_ = 6.20, and GM_*offset*(*cRutin–mRutin*)_ = 6.54. Furthermore, ΔFBG exhibited a significant positive correlation with the changes in the gut microbiota of the HFD+Rutin group (*R* = 0.92, *p* < 0.05, Supplementary Figure 2), suggesting that the decrease in FBG levels was significantly correlated with the dynamic changes in gut microbial communities during the treatment of rutin. Since GM_*offset*(*HFD*+*PA–HFD*+*SPSS*)_< GM_*offset*(*HFD*+*Rutin–HFD*+*SPSS*)_, and GM_*offset*(*cPA*–*mPA*)_< GM_*offset*(*cRutin–mRutin*)_, the results of these offset values and linear fit analyses suggest that rutin may be superior to PA in its ability to modulate the composition of the gut microbial communities (Figures 4J–L).

Moreover, to investigate the dynamic changes in gut microbial communities, the taxonomical compositions of gut microbial communities were profiled at the phylum and genus levels. First, nine phyla, namely *Actinobacteriota*, *Bacteroidota*, *Campilobacterota*, *Deferribacterota*, *Desulfobacterota*, *Firmicutes*, *Patescibacteria, Proteobacteria*, and *Verrucomicrobiota*, were found to be the dominant taxa in gut microbial communities. The relative abundances of *Firmicutes* and *Bacteroidota* in T2DM mice significantly increased and decreased, respectively, during the entire process in comparison with those in normal mice in the CD+SPSS group (Figures 4M, N), consistent with the changes in these two phyla in the gut microbial communities of HFD-induced obese mice (Daniel et al., 2014). The *Firmicutes*/*Bacteroidota* (F/B) ratio in the HFD+SPSS group increased in comparison with that in the CD+SPSS group (Figure 4O). After the oral gavage of PA and rutin was administered, the relative abundances of *Firmicutes* and *Bacteroidota* continually increased and decreased (Figures 4M, N), respectively, and the *F/B* ratio in the HFD+PA and HFD+Rutin groups increased (Figure 4O). In particular, the *F/B* ratio in the HFD+Rutin group was higher than that in the HFD+PA (Figure 4O). Subsequently, the dynamic changes in the taxonomical compositions of gut microbial communities between pre-intervention and post-intervention samples for PA and rutin were compared at the phylum level. As for the T2DM mice administered with PA, the relative abundances of *Firmicutes* and *Bacteroidota* decreased and increased (Figures 4P, Q), respectively, and the F/B ratio decreased (Figure 4R). Interestingly, the dynamic patterns of the relative abundances of *Firmicutes* and *Bacteroidota* and the F/B ratio were inverse in the gut microbial communities of mice intervened with rutin (Figures 4S–U). Furthermore, a total of 66 genera were identified, and seven taxa, including *Muribaculaceae*, *Bacteroides*, *Lactococcus, Lachnospiraceae_NK4A136_group*, *Clostridia_UCG-014*, *Lactobacillus*, and *Rikenellaceae_RC9_gut_group*, dominated in the gut microbial communities (Figure 4V). These results revealed that the taxonomical structures of gut microbial communities were significantly altered, coupled with the loss of BW and the decrease in FBG, with the intervention of PA and rutin.

### Identification of key gut microbiota, co-occurrence network analysis, and their interactions with BW and FBG during the intervention process of rutin and PA

In the previous section of our results, we primarily focused on the dynamic changes of taxa at the phylum and genus levels and ignored the roles of microbes with significantly altered gut microbial communities of T2DM mice during the intervention process of PA and rutin. Hence, we further identified the key microbiota, explored the interactions and associations among these microbes, and linked them with the changes of BW and FBG to elucidate the potential mechanisms of PA and rutin in reducing BW and decreasing FBG.

**FIGURE 5:**
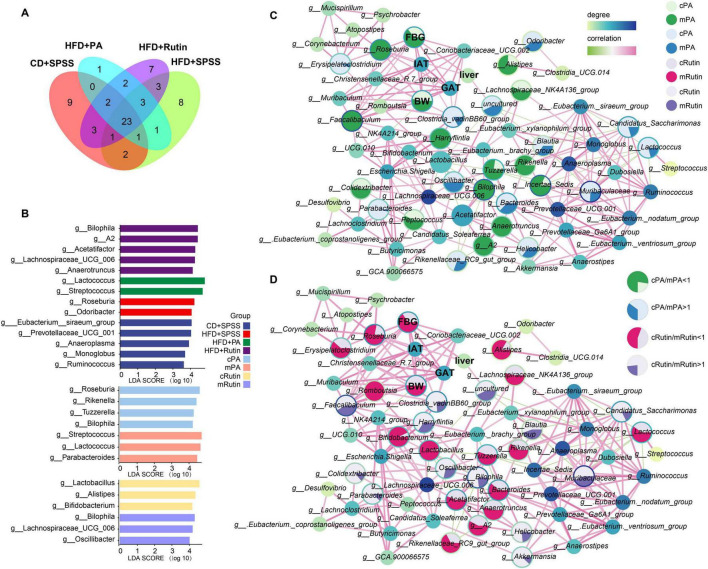
Identification of key microbes and biomarkers and construction of interaction network to reveal the potential mechanisms of PA and rutin in reducing BW and FBG. **(A)** Veen plot showed the distribution of gut microbes among groups at the genus level. **(B)** Taxonomic biomarkers for CD+SPSS, HFD+SPSS, HFD+PA, and HFD+Rutin groups and pre-intervention and post-intervention of PA and rutin were identified. Network analysis showed the interactions between gut microbes and mouse body features and the alterations of gut microbiota for the intervention with panel **(C)** PA and **(D)** rutin. The area of each circle is divided into two parts dark green and dark blue (enriched in the mPA group), and light green and light blue (enriched in the cPA group). The area of each circle is divided into two parts labeled with dark pink, dark purple (enriched in the mRutin group), and light pink and light purple (enriched in the cRutin group).

First, the distribution of core and specific genera of gut microbial communities were visualized and 23 core genera were identified during the intervention of PA and rutin (Figure 5A). Then, the significantly differed taxon at the genus level was identified among different groups with LEfSe. The results showed that *Roseburia* and *Odoribacter* harbored powerful discrimination for the HFD group, whereas *Eubacterium_siraeum_group*, *Prevotellaceae_ucg-001*, *Anaeroplasma*, *Monoglobus*, and *Ruminococcus* were suitable to the CD+SPSS group (Figure 5B). Moreover, a series of gut probiotics and short-chain-fatty-acid-producing bacteria, such as *Lactococcus*, *Streptococcus*, *Lachnospiraceae_UCG-006*, and *Anaerotruncus* were determined to be powerful discrimination taxa in the HFD+PA and HFD+Rutin groups compared with the HFD+SPSS group after the oral gavage of PA and rutin (Figure 5B and Supplementary Figure 3). Several significantly different taxa, including *Roseburia* and *Parabacteroides*, were identified in T2DM mice with the intervention of PA and rutin (Figure 5B).

Second, co-occurrence networks between the taxonomic composition of gut microbiota at the genus level and body characteristics (including BW and FBG) and the weights of three tissues were constructed at week 10 (Supplementary Figure 4). The ratio of the abundance of the same taxon and the ratio of body features between cPA and mPA groups and between cRutin and mRutin groups were calculated and visualized (Figures 5C, D), respectively. The results showed that the relative abundances of *Colidextribacter*, *Faecalibaculum*, *A2*, *Anaerotruncus*, *Tuzzerella*, *Rikenella*, *Bilophila*, *Alistipes*, *Lachnospiraceae_NK4A136_group*, and *Roseburia* in the gut microbiota communities of T2DM mice increased, and then after the oral gavage of PA, their abundances significantly decreased (all *p* < 0.05, Kruskal–Wallis test, Figure 5C). Similarly, the relative abundances of *Roseburia*, *Lactococcus*, *Bifidobacterium*, *Romboutsia*, *Tuzzerella*, *Rikenella*, *Alistipes*, *Bacteroides*, *Lactobacillus*, *Lachnospiraceae_NK4A136_group*, *Rikenellaceae_RC9_gut_group*, *A2*, and *Anaerotruncus* in the gut microbiota communities of T2DM mice increased, and then after the oral gavage of rutin, their abundances significantly decreased (all *p* < 0.05, Kruskal–Wallis test, Figure 5D). The results suggested that the relative abundances of *Roseburia*, *Tuzzerella*, *Lachnospiraceae_NK4A136_group*, *Rikenella*, *Alistipes*, *A2*, and *Anaerotruncus*, decreased after the intervention of PA and rutin. Inversely, the dynamic change patterns of *Helicobacter*, *Erysipelatoclostridium*, *Clostridia_vadinBB60_group*, *Blautia*, *Odoribacter*, *Candidatus_Saccharimonas*, *Lactococcus*, *Parabacteroides*, and *Muribaculaceae* in T2DM mice with the oral gavage of PA and those of *Parabacteroides*, *Akkermansia*, *Bilophila*, *Helicobacter*, *Clostridia_vadinBB60_group*, *Blautia*, *Muribaculaceae*, *Candidatus_Saccharimonas*, and *Oscillibacter* with the oral gavage of rutin significantly increased (all *p* < 0.05, Kruskal–Wallis test, Figures 5C, D). The abundance of *Helicobacter*, *Muribaculaceae*, *Parabacteroides*, *Clostridia_vadinBB60_group*, *Blautia*, *Candidatus_Saccharimonas*, and *Odoribacter* increased after the intervention of PA and rutin.

Third, the linkage between the alterations in mice’s body features and gut microbiota was investigated. The Pearson correlations among the gut microbiota and BW, FBG, IAT, GAT, and liver tissue were determined, and the interaction network was conducted. The results showed that *Faecalibaculum*, *Christensenellaceae_R-7_group*, *Clostridium_sensu_stricto_1*, *Coriobacteriaceae_UCG-002*, *Mucispirillum*, and *Roseburia* were significantly positively correlated with BW, whereas *NK4A214_group* was significantly negatively correlated (all *p* < 0.05, Supplementary Figure 5). Additionally, *Christensenellaceae_R-7_group*, *Clostridium_sensu_stricto_1*, *Coriobacteriaceae_UCG-002*, *Faecalibaculum*, *Mucispirillum*, and *Roseburia* were significantly positively correlated with FBG (*p* < 0.05, Supplementary Figure 5), and the relative abundance of *Roseburia* was significantly positively correlated the changes in the weight of liver tissue (*R* = 0.484, *p* < 0.05, Supplementary Figure 6). Besides, *Faecalibaculum*, *Christensenellaceae_R-7_group*, *Mucispirillum*, *Clostridium_sensu_stricto_1*, *Clostridia_vadinBB60_group*, *Coriobacteriaceae_UCG-002*, and *Roseburia*, were significantly positively correlated with the changes in IAT and GAT. In particular, *Roseburia* was strongly positively correlated with the dynamic changes in body features, including BW, FBG, IAT, GAT, and liver tissue. Our findings suggest that weight loss and FBG reduction in T2DM mice under sustained intervention with PA and rutin are strongly associated with changes in the gut microbiota.

### Identification of potential functional traits of gut microbial communities that contribute to the role of rutin and PA

**FIGURE 6:**
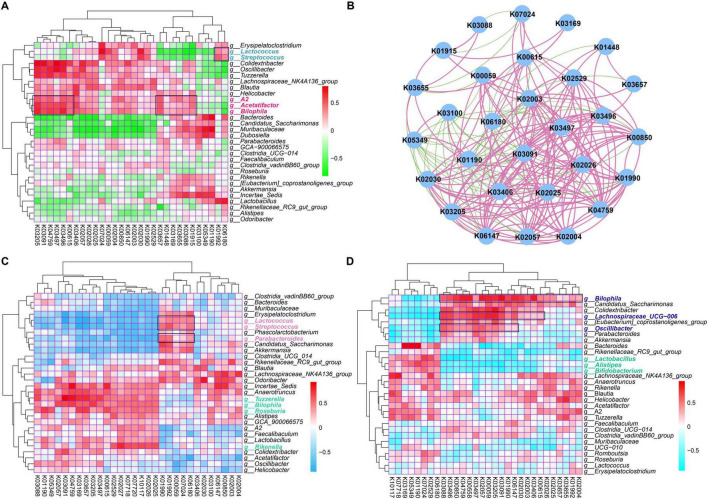
Identification of potential functional traits of gut microbial communities that contribute to the role of PA and rutin. **(A)** The heatmap showed the Spearman correlations between dominant KOs and the genus taxa based on the genus composition of gut microbial communities in CD+SPSS, HFD+SPSS, HFD+PA, and HFD+Rutin groups (week 10). **(B)** The Spearman correlations among the top 30 KOs were visualized based on the functional composition of gut microbial communities in CD+SPSS, HFD+SPSS, HFD+PA, and HFD+Rutin groups (week 10). **(C)** The Spearman correlations between dominant KOs and the genus taxa of cPA and mPA groups were determined. **(D)** The Spearman correlations between dominated KOs and the genus taxa of cRutin and mRutin groups were determined.

To obtain an in-depth understanding of the potential mechanism of PA and rutin, the functional compositions of gut microbial communities were profiled and the linkages between the dominant functional traits and key gut microbes were constructed with multiple comparisons based on Spearman’s rank correlations (Figure 6). The results showed that the associations between the discriminated taxa (biomarkers) of each group and the dominant KOs (Figure 6A) were different. Such as *Lactococcus* and *Streptococcus*, which are two biomarkers for the HFD+PA group, were significantly associated with K07024, K00059, K02030, K02004, K02003, K02529, K01992, and K06180, which are mainly involved in starch and sucrose metabolism, fatty acid metabolism, amino acid transport system, ABC transport system, *Lac*I family transcription regulatory factors, and ribosome biosynthesis (Figure 6A). Meanwhile, the biomarkers for the HFD+Rutin group, such as *Acetatifactor* and *Bilophila*, were significantly associated with abundant KOs, including K03205, K03091, K04759, K03497, K03496, K00615, K03657, K01448, K03169, K03655, K03088, and K01915, which mainly participate in the function of the secretory system, iron transport, protein signaling, *Par*B family transcription processes, chromosome segmentation proteins, pentose phosphate pathways, nucleotide excision repair, DNA replication, and amino acid metabolism (Figure 6A). Subsequently, the correlations among the top 30 KOs were calculated and visualized (Figure 6B). Then, the enrichment of KOs in the intervention of PA and rutin was compared and the functional traits of gut microbial communities were found to be mainly involved in glucose metabolism, fatty acid metabolism, amino acid metabolism, and protein transport system after PA and rutin treatment (Figures 6C, D). Overall, the results suggested that the alterations in gut microbes of the microbial communities of T2DM mice after the intervention with PA and rutin led to the enrichment of functional traits, such as the metabolism of glucose, fatty acid, and amino acids, which contributed to the role of PA and rutin in reducing BW and decreasing FBG.

## Discussion

In our present study, we verified the function of rutin in reducing weight and decreasing blood glucose levels, compared the effects between the oral gavage of PA and rutin in animal experiments, and investigated the potential mechanisms from the perspective of gut microbiota. Our results demonstrated that PA and rutin harbor the ability to lose BW and decrease FBG levels in T2DM mice, and enhance glucose tolerance and insulin sensitivity. In particular, the weight loss effect of PA was superior to that of rutin {ΔBW_(HFD+Rutin)_ = −0.59, ΔBW_(HFD+PA)_ = −3.116, and abs[ΔBW_(HFD+PA)_] > abs[ΔBW_(HFD+Rutin)_]}, whereas the FBG decrease effect of rutin was superior to that of PA {ΔFBG_(HFD+Rutin)_ = −4.56, ΔFBG_(HFD+PA)_ = −4.54, and abs[ΔFBG_(HFD+Rutin)_] > abs[ΔFBG_(HFD+PA)_]}. These results suggested that although PA and rutin harbor the ability to lose BW and decrease FBG levels, the application scenarios in which they maximize their function may differ. That is to say, the advantages of PA are rapidly reducing BW and decreasing FBG levels, and quickly controlling BW and FBG is suitable, benefiting obese populations with T2DM. Meanwhile, the advantages of rutin are slowly reducing BW and rapidly decreasing the FBG levels, benefiting patients with T2DM but without obesity. Additionally, the results of OGTT and IPITT showed that PA and rutin can enhance glucose tolerance and insulin resistance in T2DM mice, and the effects of PA are better than those of rutin.

Moreover, the histopathological results showed that the tissues of T2DM mice recovered after the oral gavage of PA and rutin, indicating the transformation of these tissues from a pathological state to a normal state. The H&E staining results of the four kinds of tissues, including PAN, liver tissue, IAT, and GAT, revealed the alterations in cells and proved that PA and rutin can improve chronic inflammation. Besides, the H&E staining results of adipose tissue sections and the measurement of liver weight indicated that PA and rutin can significantly reduce adipocyte hypertrophy. A combination of the results of PAN and liver tissue sections and the comparison results of the weight of tissues indicated that rutin is more effective in alleviating pancreatic histopathology and improving the cytopathy of liver tissue than PA.

Furthermore, previous studies have proven that the mechanism of PA in reducing BW and decreasing FBG is achieved through modulating the gut microbiota (Ferreira et al., 2023). Hence, in the present study, to elucidate the mechanism of rutin and investigate the effect variations between PA and rutin in gut microbiota remodeling, we conducted the 16S rRNA amplicon sequencing, profiled the taxonomical and functional compositions, and linked the alterations of gut microbiota with the changes of body features. Our results showed that the composition of gut microbial communities changed significantly after the oral gavage of PA or rutin. However, according to the formula offset between the two groups, we calculated the offsets of gut microbial communities for different comparative strategies and found that the changes in gut microbial communities caused by rutin were more intense than those caused by PA. Interestingly, we found the alterations in FBG were significantly positively correlated with the dynamic changes in the gut microbiota of the HFD+Rutin group (*p* < 0.05). Besides, the dynamic changes of gut microbes were explored at the phylum and genus levels. The results demonstrated that the relative abundances of *Firmicutes* and *Bacteroides* underwent significant alteration. The Firmicutes/Bacteroidetes (F/B) ratio is widely accepted to have an important influence in maintaining normal gut homeostasis (Stojanov et al., 2020). The *F/B* ratio, which is a classical index (Pammi et al., 2017), was assessed, and we found that the *F/B* ratio in the HFD+SPSS group increased compared with that in the CD+SPSS group, whereas the *F/B* ratios in the HFD+PA and HFD+Rutin groups did not decrease. Remarkably, the *F/B* ratio decreased after the oral gavage of PA and increased after the oral gavage of rutin. These results suggested that although the potential mechanisms of PA and rutin are associated with the reconstruction of gut microbiota, the affected gut microbes differ.

Besides, based on the taxonomical composition of gut microbial communities and the data of body features during the oral gavage of PA and rutin, a series of key microbes significantly linked with body features were identified, such as *Roseburia*, *Lactococcus*, *Streptococcus*, *Lachnospiraceae_UCG-006*, *Anaerotruncus*, *Bifidobacterium*, *Akkermansia*, *Bilophila*, *Faecalibaculum*, *Parabacteroides*, and *Oscillibacter*. Additionally, these key microbes were strongly correlated with several metabolic pathways, including starch and sucrose metabolism, fatty acid metabolism, amino acid transport system, and amino acid metabolism. Previous studies have reported the linkage of these taxa to T2DM and the potential mechanism was also discussed. For example, the relative abundance of *Lactococcus* in the gut microbial community was increased and negatively associated with obesity after the administration of probiotics (Kong et al., 2019). This phenomenon was observed in the HFD+PA group. Additionally, the famous gut microbiota and the next generation probiotics, *Akkermansia muciniphila*, also undergo a significant increase in the gut microbial communities after the intervention with PA in our present study, which consistent with the result in human cohort of obesity and T2DM (Zhang et al., 2021). Previous studies have shown that the relative abundance of the gut probiotics *Akkermansia* and *Lactococcus* increased, their derived metabolites, such as short-chain fatty acids (SCFAs), also significantly increased(Cai et al., 2023; Kim et al., 2023). And the function of SCFAs in the regulation of the metabolic syndrome and the maintenance of energy homeostasis and host insulin sensitivity has been confirmed (Fu et al., 2019; Liu et al., 2019). Moreover, the abundances of a series of other SCFAs-producing gut microbes, such as *Odoribacter*, *Lactococcus*, *Streptococcus*, *Lachnospiraceae*, and *Anaerotruncus* (Dai et al., 2019; Lin et al., 2020; Wang et al., 2024), were also increased. Besides, the relative abundance of *Faecalibaculum* increased significantly after the intervention of rutin, and it was strongly correlated with BW. In a previous study, the growth of *Faecalibaculum* was notably found to alleviate obesity-induced metabolic disorders by reducing the number of pro-inflammatory Thl7 cells (Kawano et al., 2022). In addition, it has been demonstrated in previous studies that PA can be catabolized and metabolized when interacting with gut microbes to produce metabolites that enhance bioactivity and bioavailability, and that these metabolites may have a positive effect on ameliorating the metabolic syndrome (Gui et al., 2023; Redondo-Castillejo et al., 2023). Rutin has also been demonstrated to alleviate colonic lesions and modulate gut microbiota in diabetic mice (Cai et al., 2023). Therefore, we speculated that PA and rutin can positively affect metabolic health in patients with T2DM and obesity by regulating the gut microbiota, affecting related metabolic pathways and metabolites, and alleviating inflammatory response. Subsequent animal experiments and human clinical trial studies still need to be conducted to explore the potential of PA and rutin in alleviating inflammation and T2DM through the modulation of gut microbiota (Mathrani et al., 2023).

## Conclusion

Our results showed that PA and rutin harbor the ability to significantly lose BW and decrease FBG levels in T2DM mice. The treatment of PA and rutin on T2DM can assist with the transformation of tissues and cells, resulting in the relief of inflammation and fat hypertrophy, and the therapeutic effects of rutin are more effective than those of PA. The results of the gut microbial community suggested that the taxonomical and functional compositions of T2DM mice administered with PA and rutin underwent significant change, and FBG exhibited a significant correlation with the offset values of gut microbial communities in rutin treatment. In addition, our findings suggest that the mechanisms of PA and rutin in reducing weight and decreasing FBG depend on the dynamic changes in several key microbes, such as *Akkermansia*, *Lactococcus*, *Odoribacter*, *Faecalibaculum*, and *Roseburia*; these microbes were significantly associated with the changes in BW and FBG, and the changes in functional traits, such as sucrose metabolism, fatty acid metabolism, amino acid transport system, and amino acid metabolism. Although PA and rutin could lower BW and reduce FBG, the application scenarios in which they maximize their function may differ, and the underlying mechanisms involve the regulation of the gut microbiota in T2DM mice. Our results provide new insights into the clinical management and treatment of BW and FBG in patients with obesity and T2DM from the perspective of gut microbiota and dietary supplements.

## Data Availability

Sequencing datasets for 30 fecal samples in our study have been deposited into NCBI’s Sequence Read Archive (SRA) database with the BioProject number: PRJNA961702.

## References

[B1] Asemi Z. Alizadeh S. A. Ahmad K. Goli M. Esmaillzadeh A. (2016). Effects of beta-carotene fortified synbiotic food on metabolic control of patients with type 2 diabetes mellitus: a double-blind randomized cross-over controlled clinical trial. *Clin. Nutr.* 35 819–825. 10.1016/j.clnu.2015.07.009 26209256

[B2] Bazyar H. Moradi L. Zaman F. Zare Javid A. (2023). The effects of rutin flavonoid supplement on glycemic status, lipid profile, atherogenic index of plasma, brain-derived neurotrophic factor (BDNF), some serum inflammatory, and oxidative stress factors in patients with type 2 diabetes mellitus: a double-blind, placebo-controlled trial. *Phytother. Res.* 37 271–284. 10.1002/ptr.7611 36101997

[B3] Bertoia M. L. Rimm E. B. Mukamal K. J. Hu F. B. Willett W. C. Cassidy A. (2016). Dietary flavonoid intake and weight maintenance: three prospective cohorts of 124,086 US men and women followed for up to 24 years. *BMJ* 352:i17. 10.1136/bmj.i17 26823518 PMC4730111

[B4] Bokulich N. A. Kaehler B. D. Rideout J. R. Dillon M. Bolyen E. Knight R. (2018). Optimizing taxonomic classification of marker-gene amplicon sequences with QIIME 2’s q2-feature-classifier plugin. *Microbiome* 6:90. 10.1186/s40168-018-0470-z 29773078 PMC5956843

[B5] Cai C. Cheng W. Shi T. Liao Y. Zhou M. Liao Z. (2023). Rutin alleviates colon lesions and regulates gut microbiota in diabetic mice. *Sci. Rep.* 13:4897. 10.1038/s41598-023-31647-z 36966186 PMC10039872

[B6] Campos F. Peixoto A. F. Fernandes P. A. R. Coimbra M. A. Mateus N. de Freitas V. (2021). The antidiabetic effect of grape pomace polysaccharide-polyphenol complexes. *Nutrients* 13:4495. 10.3390/nu13124495 34960047 PMC8709276

[B7] Chen L. Gnanaraj C. Arulselvan P. El-Seedi H. Teng H. (2019). A review on advanced microencapsulation technology to enhance bioavailability of phenolic compounds: based on its activity in the treatment of Type 2 Diabetes. *Trends Food Sci. Tech.* 85 149–162. 10.1016/j.tifs.2018.11.026

[B8] Cheng Y. Yu X. Zhang J. Chang Y. Xue M. Li X. (2019). Pancreatic kallikrein protects against diabetic retinopathy in KK Cg-A(y)/J and high-fat diet/streptozotocin-induced mouse models of type 2 diabetes. *Diabetologia* 62 1074–1086. 10.1007/s00125-019-4838-9 30838453 PMC6509079

[B9] Choy Y. Y. Jaggers G. K. Oteiza P. I. Waterhouse A. L. (2013). Bioavailability of intact proanthocyanidins in the rat colon after ingestion of grape seed extract. *J. Agric. Food Chem.* 61 121–127. 10.1021/jf301939e 23244439

[B10] Dai S. Pan M. El-Nezami H. S. Wan J. M. F. Wang M. F. Habimana O. (2019). Effects of lactic acid bacteria-fermented soymilk on isoflavone metabolites and short-chain fatty acids excretion and their modulating effects on gut microbiota. *J. Food Sci.* 84 1854–1863. 10.1111/1750-3841.14661 31206699

[B11] Daniel H. Gholami A. M. Berry D. Desmarchelier C. Hahne H. Loh G. (2014). High-fat diet alters gut microbiota physiology in mice. *ISME J.* 8 295–308. 10.1038/ismej.2013.155 24030595 PMC3906816

[B12] de Vos W. M. Tilg H. Van Hul M. Cani P. D. (2022). Gut microbiome and health: mechanistic insights. *Gut* 71 1020–1032. 10.1136/gutjnl-2021-326789 35105664 PMC8995832

[B13] Delzenne N. M. Cani P. D. Everard A. Neyrinck A. M. Bindels L. B. (2015). Gut microorganisms as promising targets for the management of type 2 diabetes. *Diabetologia* 58 2206–2217. 10.1007/s00125-015-3712-7 26224102

[B14] Delzenne N. M. Neyrinck A. M. Bäckhed F. Cani P. D. (2011). Targeting gut microbiota in obesity: effects of prebiotics and probiotics. *Nat. Rev. Endocrinol.* 7 639–646. 10.1038/nrendo.2011.126 21826100

[B15] DeMarco V. G. Aroor A. R. Sowers J. R. (2014). The pathophysiology of hypertension in patients with obesity. *Nat. Rev. Endocrinol.* 10 364–376. 10.1038/nrendo.2014.44 24732974 PMC4308954

[B16] Dhatt G. S. Agarwal M. M. Othman Y. Nair S. C. (2011). Performance of the Roche Accu-Chek active glucose meter to screen for gestational diabetes mellitus using fasting capillary blood. *Diabetes Technol. Ther.* 13 1229–1233. 10.1089/dia.2011.0097 21864017

[B17] Donath M. Y. Shoelson S. E. (2011). Type 2 diabetes as an inflammatory disease. *Nat. Rev. Immunol.* 11 98–107. 10.1038/nri2925 21233852

[B18] Douglas G. M. Maffei V. J. Zaneveld J. R. Yurgel S. N. Brown J. R. Taylor C. M. (2020). PICRUSt2 for prediction of metagenome functions. *Nat. biotechnol.* 38 685–688. 10.1038/s41587-020-0548-6 32483366 PMC7365738

[B19] Eslam M. Newsome P. N. Sarin S. K. Anstee Q. M. Targher G. Romero-Gomez M. (2020). A new definition for metabolic dysfunction-associated fatty liver disease: an international expert consensus statement. *J. Hepatol.* 73 202–209. 10.1016/j.jhep.2020.03.039 32278004

[B20] Fan Y. Pedersen O. (2021). Gut microbiota in human metabolic health and disease. *Nat. Rev. Microbiol.* 19 55–71. 10.1038/s41579-020-0433-9 32887946

[B21] Ferreira Y. A. M. Jamar G. Estadella D. Pisani L. P. (2023). Proanthocyanidins in grape seeds and their role in gut microbiota-white adipose tissue axis. *Food Chem.* 404:134405. 10.1016/j.foodchem.2022.134405 36444031

[B22] Fryk E. Olausson J. Mossberg K. Strindberg L. Schmelz M. Brogren H. (2021). Hyperinsulinemia and insulin resistance in the obese may develop as part of a homeostatic response to elevated free fatty acids: a mechanistic case-control and a population-based cohort study. *Ebiomedicine* 65:103264. 10.1016/j.ebiom.2021.103264 33712379 PMC7992078

[B23] Fu X. Liu Z. Zhu C. Mou H. Kong Q. (2019). Nondigestible carbohydrates, butyrate, and butyrate-producing bacteria. *Crit. Rev. Food Sci. Nutr.* 59 S130–S152. 10.1080/10408398.2018.1542587 30580556

[B24] Ghorbani A. (2017). Mechanisms of antidiabetic effects of flavonoid rutin. *Biomed. Pharmacother.* 96 305–312. 10.1016/j.biopha.2017.10.001 29017142

[B25] Gonzalez-Abuin N. Pinent M. Casanova-Marti A. Arola L. Blay M. Ardevol A. (2015). Procyanidins and their healthy protective effects against type 2 diabetes. *Curr. Med. Chem.* 22 39–50. 10.2174/0929867321666140916115519 25245512

[B26] Gu L. Kelm M. A. Hammerstone J. F. Beecher G. Holden J. Haytowitz D. (2004). Concentrations of proanthocyanidins in common foods and estimations of normal consumption. *J. Nutr.* 134 613–617. 10.1093/jn/134.3.613 14988456

[B27] Gui H. Sun L. Liu R. Si X. Li D. Wang Y. (2023). Current knowledge of anthocyanin metabolism in the digestive tract: absorption, distribution, degradation, and interconversion. *Crit. Rev. Food Sci. Nutr.* 63 5953–5966. 10.1080/10408398.2022.2026291 35057688

[B28] Gullón B. Lú-Chau T. A. Moreira M. T. Lema J. M. Eibes G. (2017). Rutin: a review on extraction, identification and purification methods, biological activities and approaches to enhance its bioavailability. *Trends Food Sci. Tech* 67 220–235. 10.1016/j.tifs.2017.07.008

[B29] Hall M. Beiko R. G. (2018). 16S rRNA gene analysis with QIIME2. *Methods Mol. Biol. (Clifton, N.J.)* 1849 113–129. 10.1007/978-1-4939-8728-3_8 30298251

[B30] Hameed A. Galli M. Adamska-Patruno E. Krȩtowski A. Ciborowski M. (2020). Select polyphenol-rich berry consumption to defer or deter diabetes and diabetes-related complications. *Nutrients* 12:2538. 10.3390/nu12092538 32825710 PMC7551116

[B31] Han M. Huang Y. Gui H. Xiao Y. He M. Liu J. (2022). Dynamic changes in host immune system and gut microbiota are associated with the production of SARS-CoV-2 antibodies. *Gut* 72 1996–1999. 10.1136/gutjnl-2022-327561 36207022 PMC10511961

[B32] Hopkins B. D. Goncalves M. D. Cantley L. C. (2016). Obesity and cancer mechanisms: cancer metabolism. *J. Clin. Oncol.* 34 4277–4283. 10.1200/jco.2016.67.9712 27903152 PMC5562429

[B33] Hosomi K. Saito M. Park J. Murakami H. Shibata N. Ando M. (2022). Oral administration of Blautia wexlerae ameliorates obesity and type 2 diabetes via metabolic remodeling of the gut microbiota. *Nat. Commun.* 13:4477. 10.1038/s41467-022-32015-7 35982037 PMC9388534

[B34] Jeyaraman M. M. Al-Yousif N. S. H. Singh Mann A. Dolinsky V. W. Rabbani R. Zarychanski R. (2020). Resveratrol for adults with type 2 diabetes mellitus. *Cochr. Datab. Syst. Rev.* 1:Cd011919. 10.1002/14651858.CD011919.pub2 31978258 PMC6984411

[B35] Jiao W. Sang Y. Wang X. Wang S. (2023). Metabonomics and the gut microbiome analysis of the effect of 6-shogaol on improving obesity. *Food Chem.* 404:134734. 10.1016/j.foodchem.2022.134734 36327507

[B36] Kamalakkannan N. Prince P. S. (2006). Antihyperglycaemic and antioxidant effect of rutin, a polyphenolic flavonoid, in streptozotocin-induced diabetic wistar rats. *Basic Clin. Pharmacol. Toxicol.* 98 97–103. 10.1111/j.1742-7843.2006.pto_241.x 16433898

[B37] Kawano Y. Edwards M. Huang Y. Bilate A. M. Araujo L. P. Tanoue T. (2022). Microbiota imbalance induced by dietary sugar disrupts immune-mediated protection from metabolic syndrome. *Cell* 185 3501.e–3519.e. 10.1016/j.cell.2022.08.005 36041436 PMC9556172

[B38] Kim W. K. Min S. G. Kwon H. Park S. Jo M. J. Ko G. (2023). Lactobacillus rhamnosus KBL2290 ameliorates gut inflammation in a mouse model of dextran sulfate sodium-induced colitis. *J. Microbiol. (Seoul, Korea)* 61 673–682. 10.1007/s12275-023-00061-5 37314676

[B39] Kim Y. A. Keogh J. B. Clifton P. M. (2018). Probiotics, prebiotics, synbiotics and insulin sensitivity. *Nutr. Res. Rev.* 31 35–51. 10.1017/s095442241700018x 29037268

[B40] Kong C. Gao R. Yan X. Huang L. Qin H. (2019). Probiotics improve gut microbiota dysbiosis in obese mice fed a high-fat or high-sucrose diet. *Nutrition* 60 175–184. 10.1016/j.nut.2018.10.002 30611080

[B41] Ley R. E. Turnbaugh P. J. Klein S. Gordon J. I. (2006). Microbial ecology: human gut microbes associated with obesity. *Nature* 444 1022–1023. 10.1038/4441022a 17183309

[B42] Lin R. Sun Y. Mu P. Zheng T. Mu H. Deng F. (2020). Lactobacillus rhamnosus GG supplementation modulates the gut microbiota to promote butyrate production, protecting against deoxynivalenol exposure in nude mice. *Biochem. Pharmacol.* 175:113868. 10.1016/j.bcp.2020.113868 32088259

[B43] Liu C. S. Liang X. Wei X. H. Jin Z. Chen F. L. Tang Q. F. (2019). Gegen Qinlian decoction treats diarrhea in piglets by modulating gut microbiota and short-chain fatty acids. *Front. Microbiol.* 10:825. 10.3389/fmicb.2019.00825 31057525 PMC6482297

[B44] Liu M. Huang B. Wang L. Lu Q. Liu R. (2022). Peanut skin procyanidins ameliorate insulin resistance via modulation of gut microbiota and gut barrier in type 2 diabetic mice. *J.Sci. Food Agric.* 102 5935–5947. 10.1002/jsfa.11945 35442513

[B45] Liu Q. Pan R. Ding L. Zhang F. Hu L. Ding B. (2017). Rutin exhibits hepatoprotective effects in a mouse model of non-alcoholic fatty liver disease by reducing hepatic lipid levels and mitigating lipid-induced oxidative injuries. *Int. Immunopharmacol.* 49 132–141. 10.1016/j.intimp.2017.05.026 28577437

[B46] Liu W. Zhao S. Wang J. Shi J. Sun Y. Wang W. (2017). Grape seed proanthocyanidin extract ameliorates inflammation and adiposity by modulating gut microbiota in high-fat diet mice. *Mol. Nutr. Food Res.* 61 10.1002/mnfr.201601082 28500724

[B47] Mathrani A. Yip W. Sequeira-Bisson I. R. Barnett D. Stevenson O. Taylor M. W. (2023). Effect of a 12-week polyphenol rutin intervention on markers of pancreatic β-cell function and gut microbiota in adults with overweight without diabetes. *Nutrients* 15:3360. 10.3390/nu15153360 37571297 PMC10420824

[B48] Ogurtsova K. da Rocha Fernandes J. D. Huang Y. Linnenkamp U. Guariguata L. Cho N. H. (2017). IDF diabetes atlas: global estimates for the prevalence of diabetes for 2015 and 2040. *Diabetes Res. Clin. Pract.* 128 40–50. 10.1016/j.diabres.2017.03.024 28437734

[B49] Ola M. S. Ahmed M. M. Ahmad R. Abuohashish H. M. Al-Rejaie S. S. Alhomida A. S. (2015). Neuroprotective effects of rutin in streptozotocin-induced diabetic rat retina. *J. Mol. Neurosci.* 56 440–448. 10.1007/s12031-015-0561-2 25929832

[B50] Pammi M. Cope J. Tarr P. I. Warner B. B. Morrow A. L. Mai V. (2017). Intestinal dysbiosis in preterm infants preceding necrotizing enterocolitis: a systematic review and meta-analysis. *Microbiome* 5:31. 10.1186/s40168-017-0248-8 28274256 PMC5343300

[B51] Pang X. X. Bai Q. Wu F. Chen G. J. Zhang A. H. Tang C. S. (2016). Urotensin II induces ER stress and EMT and increase extracellular matrix production in renal tubular epithelial cell in early diabetic mice. *Kidney Blood Pressure Res.* 41 434–449. 10.1159/000443445 27467277

[B52] Prevel R. Enaud R. Orieux A. Camino A. Berger P. Boyer A. (2022). Gut bacteriobiota and mycobiota are both associated with Day-28 mortality among critically ill patients. *Crit. Care* 26:105. 10.1186/s13054-022-03980-8 35418098 PMC9007252

[B53] Prior R. L. Gu L. (2005). Occurrence and biological significance of proanthocyanidins in the American diet. *Phytochemistry* 66 2264–2280. 10.1016/j.phytochem.2005.03.025 15904940

[B54] Redondo-Castillejo R. Garcimartín A. Hernández-Martín M. López-Oliva M. E. Bocanegra A. Macho-González A. (2023). Proanthocyanidins: impact on gut microbiota and intestinal action mechanisms in the prevention and treatment of metabolic syndrome. *Int. J. Mol. Sci.* 24:5369. 10.3390/ijms24065369 36982444 PMC10049473

[B55] Salvadó M. J. Casanova E. Fernández-Iglesias A. Arola L. Bladé C. (2015). Roles of proanthocyanidin rich extracts in obesity. *Food Funct.* 6 1053–1071. 10.1039/c4fo01035c 25669490

[B56] Segata N. Izard J. Waldron L. Gevers D. Miropolsky L. Garrett W. S. (2011). Metagenomic biomarker discovery and explanation. *Genome Biol.* 12:R60. 10.1186/gb-2011-12-6-r60 21702898 PMC3218848

[B57] Sonnenburg J. L. Bäckhed F. (2016). Diet-microbiota interactions as moderators of human metabolism. *Nature* 535 56–64. 10.1038/nature18846 27383980 PMC5991619

[B58] Stojanov S. Berlec A. Štrukelj B. (2020). The influence of probiotics on the firmicutes/bacteroidetes ratio in the treatment of obesity and inflammatory bowel disease. *Microorganisms* 8:1715. 10.3390/microorganisms8111715 33139627 PMC7692443

[B59] Tomás-Barberán F. A. Selma M. V. Espín J. C. (2016). Interactions of gut microbiota with dietary polyphenols and consequences to human health. *Curr. Opin. Clin. Nutr. Metab. Care* 19 471–476. 10.1097/mco.0000000000000314 27490306

[B60] Tung Y. T. Zeng J. L. Ho S. T. Xu J. W. Li S. Wu J. H. (2021). Anti-NAFLD effect of Djulis hull and its major compound, rutin, in mice with High-Fat Diet (HFD)-induced obesity. *Antioxidants (Basel)* 10:1694. 10.3390/antiox10111694 34829565 PMC8615009

[B61] Valentová K. Vrba J. Bancířová M. Ulrichová J. Křen V. (2014). Isoquercitrin: pharmacology, toxicology, and metabolism. *Food Chem. Toxicol.* 68 267–282. 10.1016/j.fct.2014.03.018 24680690

[B62] Wang Q. Zhang Y. Lu R. Zhao Q. Gao Y. (2024). The multiple mechanisms and therapeutic significance of rutin in metabolic dysfunction-associated fatty liver disease (MAFLD). *Fitoterapia* 178:106178. 10.1016/j.fitote.2024.106178 39153555

[B63] Wen J. J. Li M. Z. Chen C. H. Hong T. Yang J. R. Huang X. J. (2023). Tea polyphenol and epigallocatechin gallate ameliorate hyperlipidemia via regulating liver metabolism and remodeling gut microbiota. *Food Chem.* 404:134591. 10.1016/j.foodchem.2022.134591 36444016

[B64] World Health Organization. (2021). *Obesity and Overweight.* Geneva: World Health Organization.

[B65] Yang G. Wei J. Liu P. Zhang Q. Tian Y. Hou G. (2021). Role of the gut microbiota in type 2 diabetes and related diseases. *Metabolism* 117:154712. 10.1016/j.metabol.2021.154712 33497712

[B66] Yao M. J. Teng H. Lv Q. Gao H. Guo T. Lin Y. (2021). Anti-hyperglycemic effects of dihydromyricetin in streptozotocin-induced diabetic rats. *Food Sci. Hum. Well* 10 155–162. 10.1016/j.fshw.2021.02.004

[B67] Yeh C. H. Yang J. J. Yang M. L. Li Y. C. Kuan Y. H. (2014). Rutin decreases lipopolysaccharide-induced acute lung injury via inhibition of oxidative stress and the MAPK-NF-κB pathway. *Free Radic. Biol. Med.* 69 249–257. 10.1016/j.freeradbiomed.2014.01.028 24486341

[B68] Yoo J. Y. Kim S. S. (2016). Probiotics and prebiotics: present status and future perspectives on metabolic disorders. *Nutrients* 8:173. 10.3390/nu8030173 26999199 PMC4808900

[B69] Yu J. Dong R. Da J. Li J. Yu F. Zha Y. (2019). High-mobility group nucleosome-binding protein 1 mediates renal fibrosis correlating with macrophages accumulation and epithelial-to-mesenchymal transition in diabetic nephropathy mice model. *Kidney Blood Pressure Res.* 44 331–343. 10.1159/000499877 31203283

[B70] Yuan X. Wei G. You Y. Huang Y. Lee H. J. Dong M. (2017). Rutin ameliorates obesity through brown fat activation. *FASEB J.* 31 333–345. 10.1096/fj.201600459RR 28049156

[B71] Yusuf S. Hawken S. Ounpuu S. Bautista L. Franzosi M. G. Commerford P. (2005). Obesity and the risk of myocardial infarction in 27,000 participants from 52 countries: a case-control study. *Lancet* 366 1640–1649. 10.1016/s0140-6736(05)67663-5 16271645

[B72] Zeng Y. X. Wang S. Wei L. Cui Y. Y. Chen Y. H. (2020). Proanthocyanidins: components, pharmacokinetics and biomedical properties. *Am. J. Chin. Med.* 48 813–869. 10.1142/s0192415x2050041x 32536248

[B73] Zhang C. Ma S. Wu J. Luo L. Qiao S. Li R. (2020). A specific gut microbiota and metabolomic profiles shifts related to antidiabetic action: the similar and complementary antidiabetic properties of type 3 resistant starch from Canna edulis and metformin. *Pharmacol. Res.* 159:104985. 10.1016/j.phrs.2020.104985 32504839

[B74] Zhang J. Ni Y. Qian L. Fang Q. Zheng T. Zhang M. (2021). Decreased abundance of akkermansia muciniphila leads to the impairment of insulin secretion and glucose homeostasis in lean type 2 diabetes. *Adv. Sci.* 8:e2100536. 10.1002/advs.202100536 34085773 PMC8373164

[B75] Zhao Z. Chen Y. Li X. Zhu L. Wang X. Li L. (2022). Myricetin relieves the symptoms of type 2 diabetes mice and regulates intestinal microflora. *Biomed. Pharmacother.* 153:113530. 10.1016/j.biopha.2022.113530 36076610

