# Supplementary figures and images for: Dietary supplementation with proanthocyanidins and rutin alleviates the symptoms of type 2 diabetes mice and regulates gut microbiota
Source: Front Microbiol. 2025 Jan 6;15:1513935. doi: 10.3389/fmicb.2024.1513935 (PMC11743507; doi:10.3389/fmicb.2024.1513935)

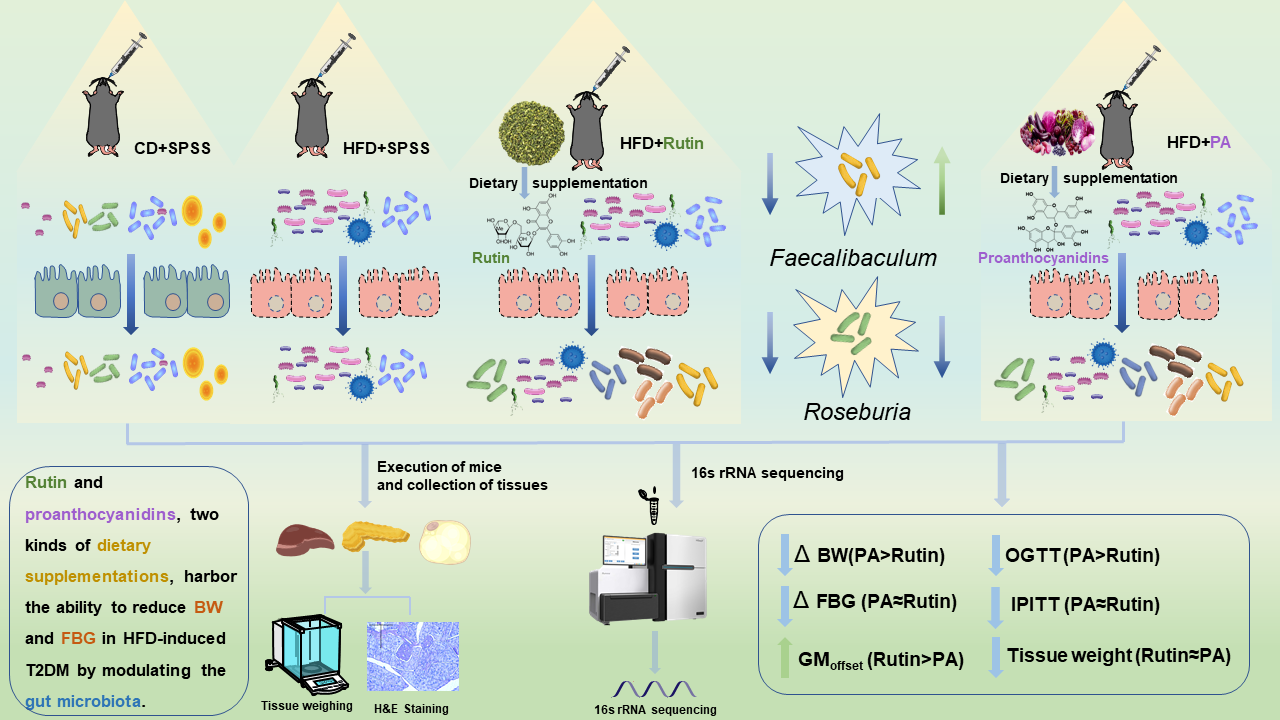

Supplement: Supplementary file 2 [file Image_1.png]
