# Supplementary material for: Dietary supplementation with proanthocyanidins and rutin alleviates the symptoms of type 2 diabetes mice and regulates gut microbiota
Source: Front Microbiol. 2025 Jan 6;15:1513935. doi: 10.3389/fmicb.2024.1513935 (PMC11743507; doi:10.3389/fmicb.2024.1513935)
Supplement: Supplementary file 3 [file Data_Sheet_1.docx]

**Supplementary Material**

**Dietary supplementation with proanthocyanidins and rutin alleviates the symptoms of type 2 diabetes mice and regulates gut microbiota**

Yue Gao^1, 2, 3, †^, Binbin Huang^4, †^, Yunyi Qin^1^, Bing Qiao^1^, Mengfei Ren^1^, Liqing Cao^1^, Yan Zhang^2, *^, Maozhen Han^1, 3, *^

^1^ School of Life Sciences, Anhui Medical University, Hefei, Anhui 230032, China.

^2^ School of Life Sciences, Hefei Normal University, Hefei, Anhui 230601, China.

^3^ Microbial medicinal resources development research team, Anhui Provincial Institute of Translational Medicine, Hefei, Anhui 230032, China.

^4^ School of Public Health, Anhui Medical University, Hefei, Anhui 230032, China.

†These authors contributed equally to this work.

^*^ Corresponding author E-mail: hanmz@ahmu.edu.cn; zhangyanwind@163.com

**1. Abbreviations used in this study**

**2. Supplementary figures**

**Fig. S1.** The results of sparsity curve analysis.

**Fig. S2.** Linear fit of ΔFBG and GM _offset(HFD+Rutin-HFD)_ in the HFD+Rutin group.

**Fig. S3.** LEfSe-identified biomarkers were identified as genus and visualized to display the alterations in the taxonomic composition of key bacterial groups of the gut microbial community at the genus level.

**Fig. S4.** The interactions among gut microbiota, BW, FBG, liver, IAT and GAT in mice at week 10.

**Fig. S5.** Linear fit of body characteristics (including BW and FBG) to gut microbes.

**Fig. S6.** The correlations between the dynamic alterations of gut microbes and the changes of tissues.

**1. Abbreviations used in this study**

| FBG | fasting blood glucose |
| --- | --- |
| HFD | high-fat diets |
| T2DM | type 2 diabetes |
| BW | body weight |
| PA | proanthocyanidin |
| OGTT | oral glucose tolerance tests |
| IPITT | intraperitoneal glucose tolerance tests |
| PAN | pancreatic |
| H&E | hematoxylin and eosin |
| CD | chow diet |
| SPSS | stroke-physiological saline solution |
| AUC | area under the curve |
| IAT | inguinal adipose tissue |
| GAT | gonadal adipose tissue |
| PBS | phosphate-buffered saline |
| LEfSe | Linear discriminate analysis effect size |
| PCoA | principal coordinate analysis |
| GM_offset_ | offset of the gut microbial communities |
| mPA | PA treatment week 4 |
| cPA | PA treatment week 10 |
| mRutin | Rutin treatment week 4 |
| cRutin | Rutin treatment week 10 |
| △ | Bacteria and body phenotype data were positively correlated. |
| ▽ | Bacteria and body phenotype data were negatively correlated. |


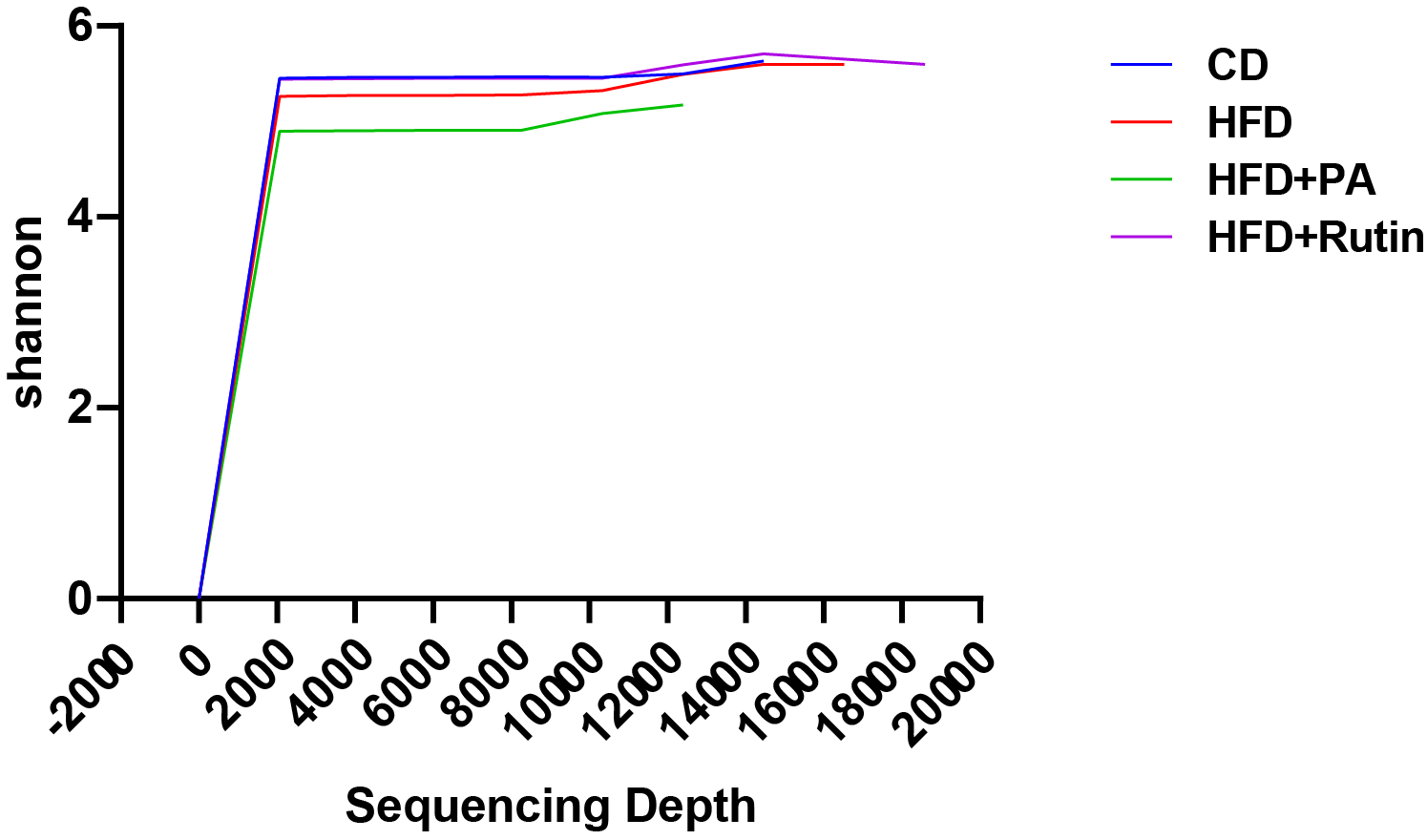


**Fig. S1.** **The results of sparsity curve analysis.** The results of sparsity curve analysis suggested that the sequencing depth is enough and the majority of microbes of each sample were capture.


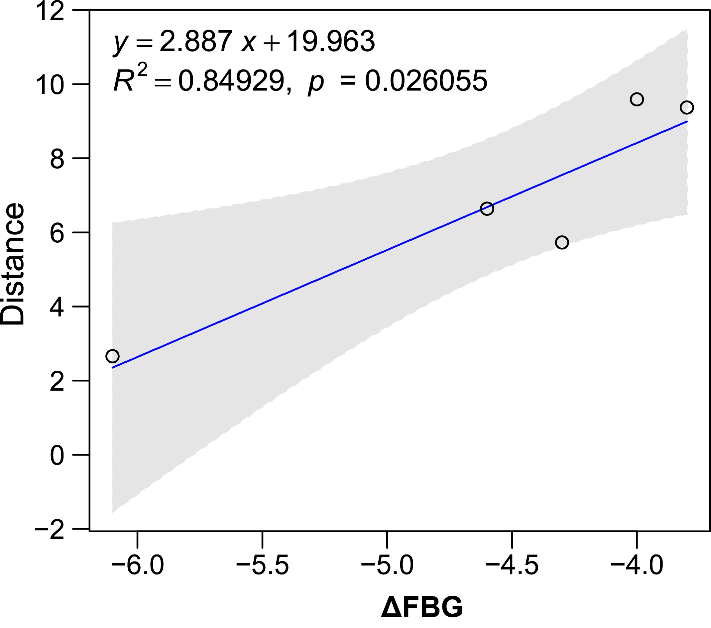


**Fig. S2. Linear fit of** **ΔFBG and GM _offset(HFD+Rutin-HFD)_ in the HFD+Rutin group.** ΔFBG of HFD+Rutin group has a significant positive correlation with GM _offset(HFD+Rutin-HFD)_.


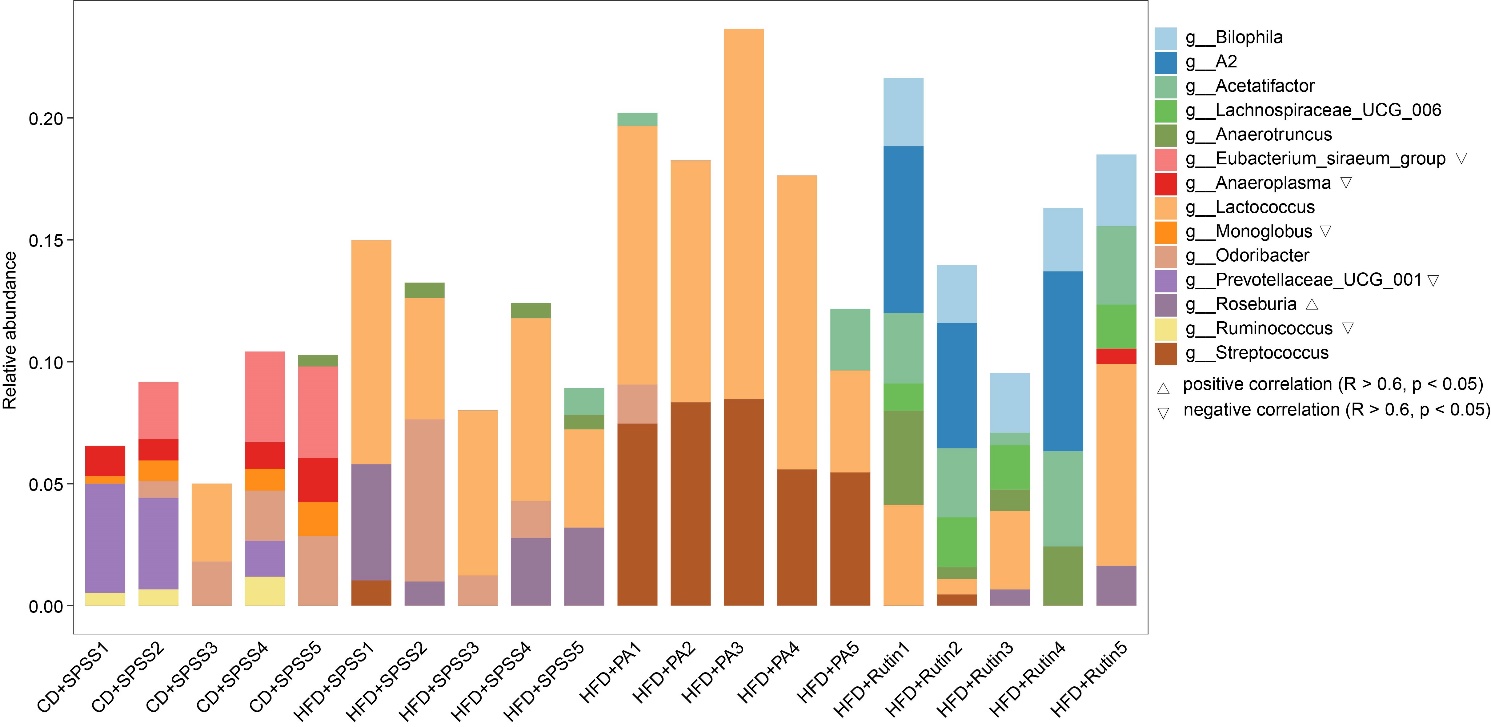


**Fig. S3.** LEfSe-identified biomarkers were identified as genus and visualized to display the alterations in the taxonomic composition of key bacterial groups of the gut microbial community at the genus level. △: Bacteria and body phenotype data were positively correlated. ▽: Bacteria and body phenotype data were negatively correlated.


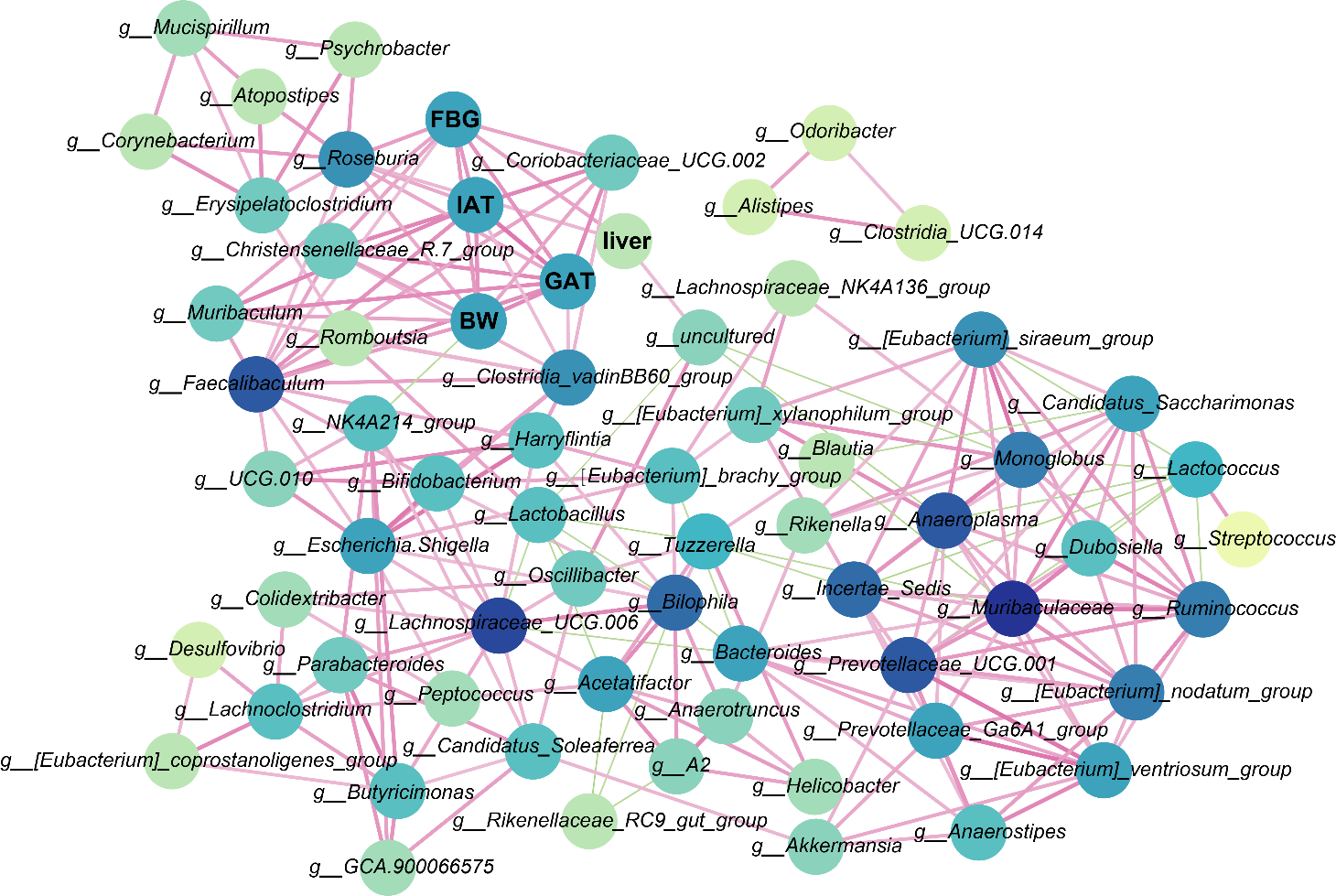


**Fig. S4. The interactions among gut microbiota, BW, FBG, liver, IAT and GAT in mice at week 10.** An interaction network between the taxonomic composition of the gut microbiota genus and physical characteristics (including BW and FBG) and weight of liver, IAT, and GAT was constructed at week 10.


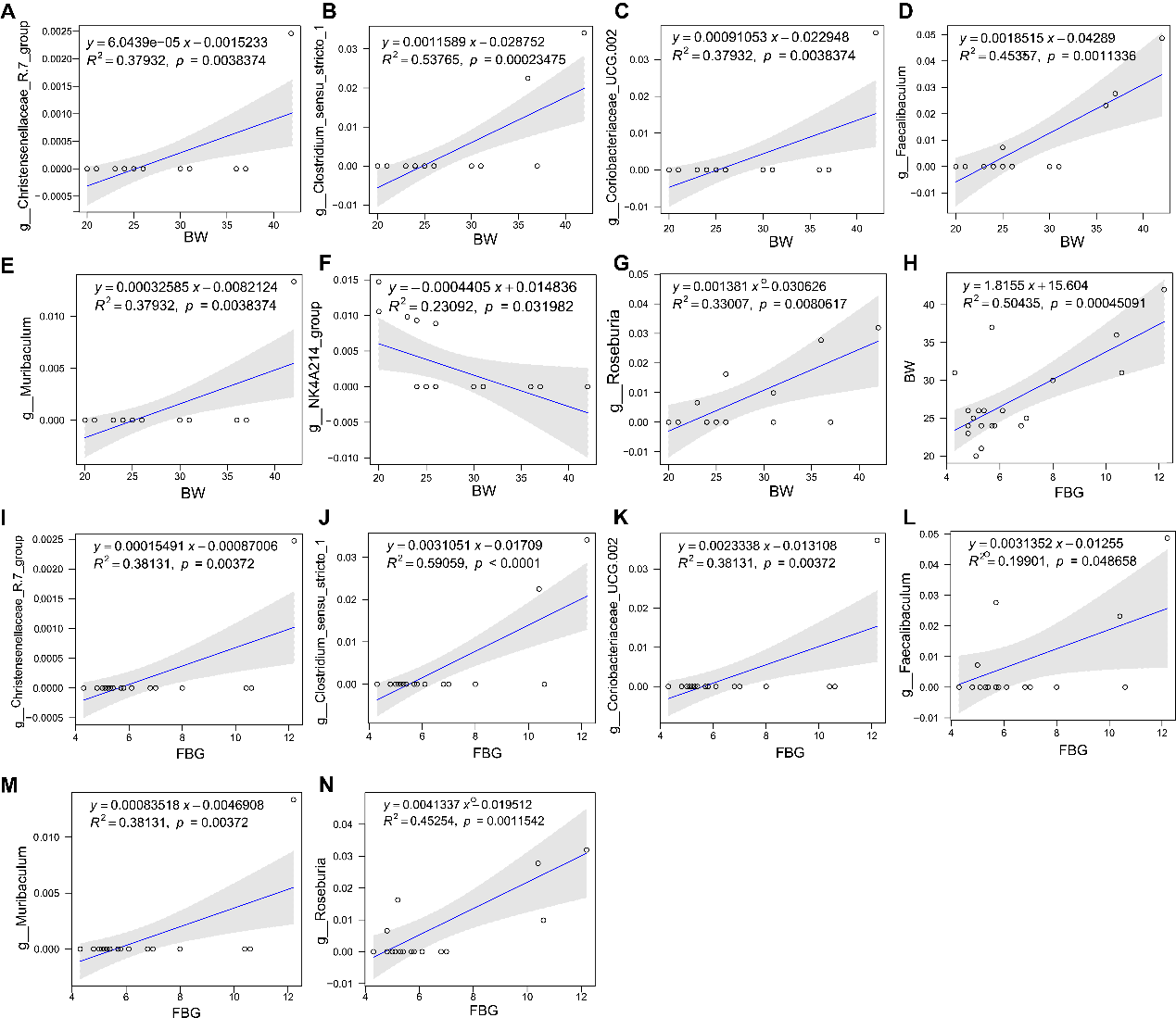


**Fig. S5. Linear fit of body characteristics (including BW and FBG) to gut microbes.** A strongly significant correlation existed in the BW values and **(A)** abundances of *g__Christensenellaceae_R-7_group*, **(B)** abundances of *g__Clostridium_sensu_stricto_1*, **(C)** abundances of *g__Coriobacteriaceae_UCG-002*, **(D)** abundances of *g__Faecalibaculum*, **(E)** abundances of *g__Muribaculum*, **(F)** abundances of *g__NK4A214_group*, **(G)** abundances of *g__Roseburia*. **(H)** A strong correlation between BW and FBG. A strongly significant correlation existed in the FBG values and **(I)** abundances of *g__Christensenellaceae_R-7_group*, **(J)** abundances of *g__Clostridium_sensu_stricto_1*, **(K)** abundances of *g__Coriobacteriaceae_UCG-002*, **(L)** abundances of *g__Faecalibaculum*, **(M)** abundances of *g__Muribaculum,* **(N)** abundances of *g__Roseburia*.


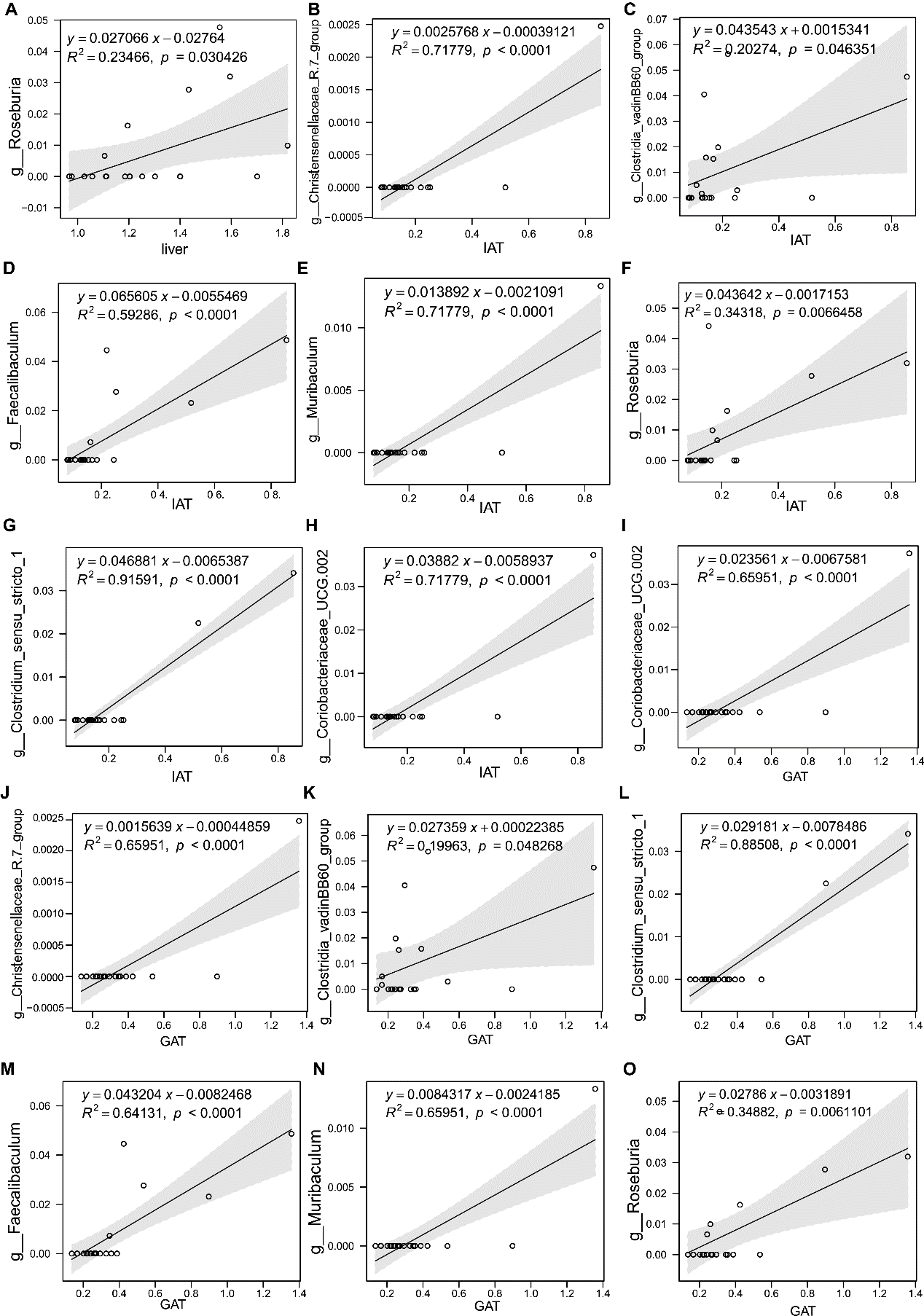


**Fig. S6. The correlations between the dynamic alterations of gut microbes and the changes of tissues.** **(A)** A strongly significant correlation existed in the liver tissue weight values and abundances of *g__Roseburia*. A strongly significant correlation existed in the IAT average weight values and **(B)** abundances of *g__Christensenellaceae_R-7_group*, **(C)** abundances of *g__Clostridia_vadinBB60_group*, **(D)** abundances of *g__Faecalibaculum*, **(E)** abundances of *g__Muribaculum*, **(F)** abundances of *g__Roseburia*, **(G)** abundances of *g__Clostridium_sensu_stricto_1*, **(H)** abundances of *g__Coriobacteriaceae_UCG-002*. A strongly significant correlation existed in the GAT average weight values and **(I)** abundances of *g__Coriobacteriaceae_UCG-002*, **(J)** abundances of *g__Christensenellaceae_R-7_group*, **(K)** abundances of *g__Clostridia_vadinBB60_group*, **(L)** abundances of *g__Clostridium_sensu_stricto_1*, **(M)** abundances of *g__Faecalibaculum*, **(N)** abundances of *g__Muribaculum,* **(O)** abundances of *g__Roseburia*.
